# Three-dimensional topology of the SMC2/SMC4 subcomplex from chicken condensin I revealed by cross-linking and molecular modelling

**DOI:** 10.1098/rsob.150005

**Published:** 2015-02-25

**Authors:** Helena Barysz, Ji Hun Kim, Zhuo Angel Chen, Damien F. Hudson, Juri Rappsilber, Dietlind L. Gerloff, William C. Earnshaw

**Affiliations:** 1 Wellcome Trust Centre for Cell Biology, Institute of Cell Biology, University of Edinburgh, Michael Swann Building, Kings Buildings, Mayfield Road, Edinburgh EH9 3JR, UK; 2 Murdoch Children's Research Institute, Royal Children's Hospital, Parkville, Victoria 3052, Australia; 3 Department of Paediatrics, University of Melbourne, Parkville, Victoria 3052, Australia; 4 Department of Bioanalytics, Institute of Biotechnology, Technische Universität Berlin, 13355 Berlin, Germany; 5 Foundation for Applied Molecular Evolution, PO Box 13174, Gainesville, FL 32604, USA

**Keywords:** SMC, condensin, structure, cross-linking, mass spectrometry, coiled-coil

## Abstract

SMC proteins are essential components of three protein complexes that are important for chromosome structure and function. The cohesin complex holds replicated sister chromatids together, whereas the condensin complex has an essential role in mitotic chromosome architecture. Both are involved in interphase genome organization. SMC-containing complexes are large (more than 650 kDa for condensin) and contain long anti-parallel coiled-coils. They are thus difficult subjects for conventional crystallographic and electron cryomicroscopic studies. Here, we have used amino acid-selective cross-linking and mass spectrometry combined with structure prediction to develop a full-length molecular draft three-dimensional structure of the SMC2/SMC4 dimeric backbone of chicken condensin. We assembled homology-based molecular models of the globular heads and hinges with the lengthy coiled-coils modelled in fragments, using numerous high-confidence cross-links and accounting for potential irregularities. Our experiments reveal that isolated condensin complexes can exist with their coiled-coil segments closely apposed to one another along their lengths and define the relative spatial alignment of the two anti-parallel coils. The centres of the coiled-coils can also approach one another closely *in situ* in mitotic chromosomes. In addition to revealing structural information, our cross-linking data suggest that both H2A and H4 may have roles in condensin interactions with chromatin.

## 2. Introduction

Complexes containing SMC proteins play central roles in regulating key chromatin transactions during mitotic chromosome formation and segregation, in DNA repair, transcription and in partitioning the replicated genome. Although bacterial and archaeal SMC complexes typically involve homodimers [1,2], their eukaryotic counterparts consist of a heterodimer (SMC2/SMC4 in condensin, SMC1/SMC3 in cohesin and SMC5/SMC6 in the SMC5/6 complex) associated with three or more non-SMC subunits [3–8].

Eukaryotic SMC proteins have a conserved architecture, with N- and C- globular ‘head’ domains at either end and a globular ‘hinge’ in the middle. The proteins fold back on themselves to create an approximately 45 nm-long intramolecular anti-parallel coiled-coil with the hinge domain at one end and the bipartite head domain at the other [9–12]. A variety of non-SMC subunits associate with this head domain. When the N- and C-terminal head segments pair with one another they assemble an ATP-binding cassette (ABC)-type ATPase domain [9] that is characteristic for SMC proteins. The hinge domain mediates SMC protein dimerization, can bind DNA [13–15] and can transmit structural changes along the coiled-coil region to the head domains [11,16,17]. In condensin and cohesin, a wealth of evidence demonstrates that the two SMC proteins are packed side by side, with their paired hinge regions at one end and the ATPase domains at the other [18,19]. Both microscopy and biochemical analyses have suggested that cohesin can form a ring capable of embracing two chromatin fibres, whereas isolated condensin often appears to fold back on itself forming a closed rod-like structure [18,20–23]. Despite their differing appearance, recent evidence suggests that condensin may also function by encircling chromatin fibres [24].

In addition to the SMC2 and SMC4 core subunits, condensin I complexes also contain three non-SMC subunits: CAP-H, CAP-G and CAP-D2 (in condensin II these are CAP-H2, CAP-G2 and CAP-D3) [25,26]. These subunits are responsible for differences in the timing and patterns of association of condensin I and II with chromosomes [27], and also for their differing roles in chromosome structure. Condensin I is thought to be involved primarily in lateral compaction of the mitotic chromosome axes, whereas condensin II is required for the rigidity of those axes [28,29].

CAP-H is a member of the kleisin family [30] that bridges between the two paired catalytic domains of SMC2 and SMC4, with the CAP-H N-terminus binding the former and its C-terminus the latter [31]. Based on a recent crystal structure of the kleisin Scc1 associated with cohesin heads, it is possible that CAP-H may also associate with the proximal portions of the condensin coiled-coil [32]. CAP-G and CAP-D2 are both HEAT (huntingtin, elongation factor 3, protein phosphatase 2A (PP2A) and TOR1) repeat proteins [33], and a recent study [34] suggests that those repeats may be involved in DNA binding. That study presented evidence suggesting that the CAP-H/CAP-G/CAP-D2 complex is involved in efficient targeting of condensin to chromosomes and in activation of the SMC2/SMC4 ATPase. Previous published work had suggested that the non-SMC subunits of condensin are phosphorylated in mitosis [25,35], and that this phosphorylation correlates with activation of the supercoiling activity of condensin [36]. The exact role of this supercoiling activity in mitotic chromosomes remains unknown.

Efforts to obtain higher resolution structures of the various SMC-containing complexes have been hampered by the sheer size of the constituent proteins (for example, the predicted molecular mass of the pentameric condensin complex is more than 660 kDa), and also by the flexible coiled-coil structure of the SMC proteins [18,20,37]. Despite the fact that coiled-coils were among the earliest structures to be identified from amino acid sequence information [38,39], high-resolution structural analysis of coiled-coil-containing proteins remains a challenge. Long two-stranded coiled-coil segments like those predicted in condensin and cohesin [3,9] are difficult to characterize structurally by high-resolution techniques owing to their elongated shape, local intrinsic flexibility [40] and tendency to aggregate [41]. Consequently, atomic coordinates for natural coiled-coil segments are both scarce and much shorter than the estimated 300–400 residues predicted to form anti-parallel coiled-coils in SMC2 and SMC4 [42–44].

Recently, systematic amino acid-selective cross-linking coupled with mass spectrometry (CLMS) analysis has contributed important structural insights into proteins that are otherwise difficult to study [45,46]. CLMS allowed determination of the organization of the parallel coiled-coils of the kinetochore-associated NDC80 complex [47], enabling production of an NDC80 bonsai complex that was subsequently characterized by X-ray crystallography [48]. CLMS has also contributed significantly to characterization of the nuclear pore complex [49], the PP2A network [50], RNA polymerase II both in association with transcription factor IIF [51] and in the pre-initiation complex [52], the yeast SMC3/Scc1 interaction [32,53] and to mapping the interaction between microtubules and the structurally flexible Ska1 domain [54].

Here, we have used established template-based molecular modelling and a cross-link-constrained prediction strategy tailored to the characteristics of coiled-coil regions, to produce a low-resolution molecular structure of the SMC2/SMC4 core of the chicken condensin complex. Modelling of SMC2 and SMC4 head and hinge regions used several high-resolution crystal structures as templates. To model the lengthy anti-parallel coiled-coils of SMC2 and SMC4 and determine their quaternary structure at low resolution, vital constraints on the structure were revealed through analysis of 120 cross-links that were induced with bis(sulfosuccinimidyl)suberate (BS3) and mapped by mass spectrometry. Of those, 117 could be incorporated into our structure within the constraints imposed by the length of the cross-linker. The model presented here will be an important resource for future structure-informed mutagenesis and functional studies of vertebrate condensin *in vitro* and *in vivo*.

## 3. Results

### 3.1. Multiple cross-linked species of purified condensin

Our studies employed two DT40 knockout cell lines expressing epitope-tagged condensin subunits: SMC2 knockout cells expressing streptavidin-binding peptide (SBP)-tagged SMC2 and CAP-H knockout cells expressing CAP-H-SBP-GFP [29,55]. These tagged proteins are functional by the criterion of rescuing the life of the cells and are the only form of each protein expressed in the cells used.

**Figure 1.**
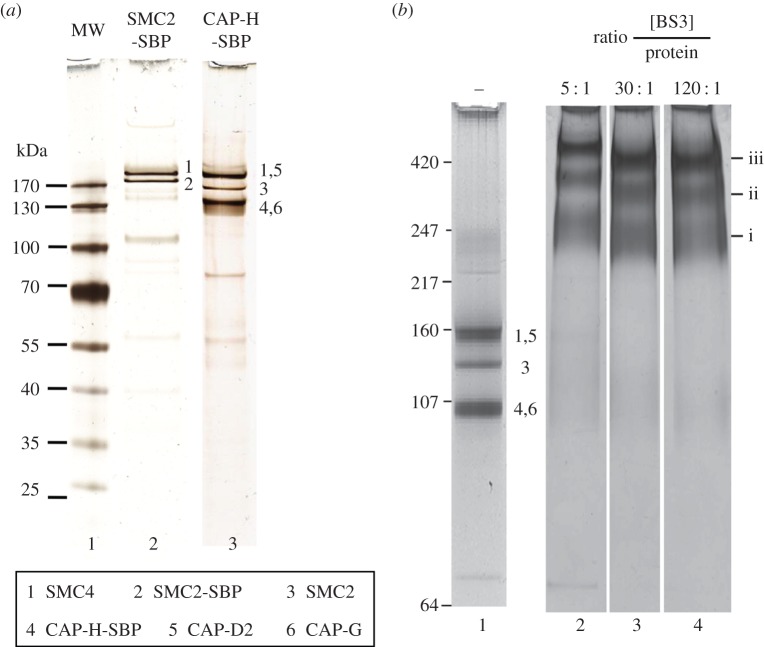
Cross-linking of isolated condensin I complex. (*a*) Composition of condensin complex purified from mitotic DT40 cells using tagged SMC2 or CAP-H. (*b*) Cross-linker titration of condensin holocomplex. A fixed amount of isolated complex (at 0.05 μg μl^−1^) was incubated with increasing amounts of BS3 cross-linker, subjected to SDS–PAGE and analysed by mass spectrometry. Based on gel mobilities, we postulate that band i represents an assortment of cross-linked dimers, band ii is likely to be cross-linked trimers and band iii is likely to be the cross-linked condensin pentamer.

When DT40 mitotic cells were used to pull down SBP-tagged SMC2, the bound material consisted largely of an SMC2 and SMC4 subcomplex, with the non-SMC subunits not visible on denaturing gels (figure 1*a*). When the pull-down was performed using SBP-tagged CAP-H kleisin subunit, CAP-H was captured together with all other subunits of the condensin holocomplex (figure 1*a*). However, a significant proportion of the SMC2 and SMC4 remained in the supernatant (data not shown). These results suggest that not all SMC2 and SMC4 in mitotic cells is present as canonical pentameric condensin complex. Indeed, Hirano *et al.* [25] identified an 8S condensin complex from *Xenopus* eggs as being composed of SMC2 and SMC4 and lacking the non-SMC subunits. Such a discrete SMC2/SMC4 complex was previously proposed to possess DNA re-annealing activity [56].

Treatment of the isolated holocomplex with the cross-linker bis(sulfosuccinimidyl)suberate (BS3), which prefers the primary amine lysine, but will also cross-link serine, threonine or tyrosine, resulted in the appearance of three new broad bands (i, ii and iii) in SDS–PAGE, regardless of the amount of cross-linker used (figure 1*b*, lanes 2–4). Mass spectrometric characterization confirmed that each band contained all five condensin subunits, which were identified with at least 50% sequence coverage. Given the remarkably similar molecular weights of four of the five condensin subunits (CAP-H is slightly smaller), we suspect that band i consists of all possible combinations of cross-linked dimers (predicted Mr ∼ 250 kDa), band ii is likely to be trimers (predicted Mr ∼ 370 kDa), and band iii is likely to be cross-linked pentamers (predicted Mr ∼ 650 kDa). It is not clear how cross-linking would affect the mobility of such large proteins in SDS–PAGE, but this explanation fits with the pattern of cross-links observed in the various bands (see below).

### 3.2. Mapping the architecture of the condensin I complex by cross-linking coupled with mass spectrometry

**Figure 2.**
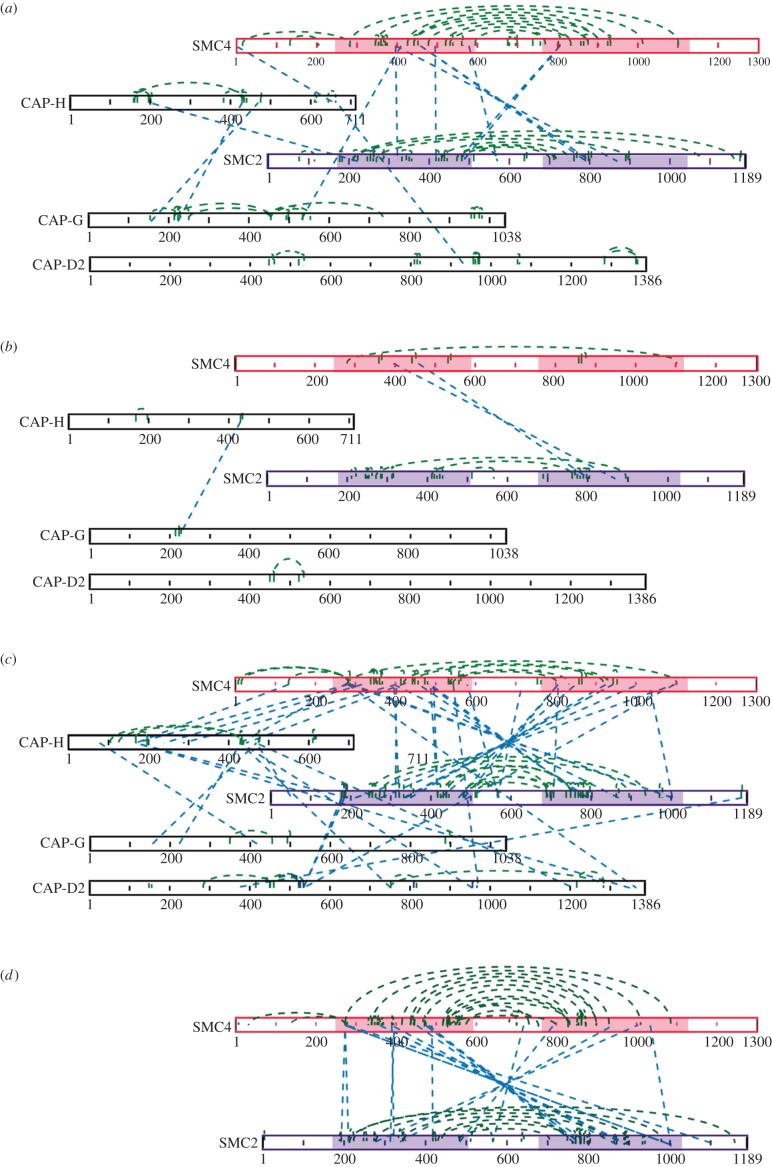
Cross-linking reveals close contacts between the SMC2 and SMC4 coiled-coil domains. Cross-link maps for (*a*) band i (*b*) band ii (*c*) band iii and (*d*) SMC2/SMC4 subcomplex visualized using xiNET (www.crosslinkviewer.org) [57]. Dashed green lines show links within subunits. Dashed blue lines show links between subunits. The coiled-coils of SMC4 are shown in red, whereas the coiled-coils of SMC2 are purple. CAP-H, CAP-G and CAP-D2 cross-link to the head and coiled-coil domains, but not to the hinges.

The three products of condensin complex cross-linking were separately investigated by mass spectrometry (figure 2). Analysis of the lowest molecular weight product (band i) yielded a total of 89 high-confidence linkage sites (see Material and methods) that could be confirmed by manual spectral analysis. All condensin cross-links identified in this analysis are listed in the electronic supplementary material, table S1.

Many cross-links were detected in the coiled-coil regions of SMC2 and SMC4. These regions are easily accessible to BS3 and contain numerous lysine residues. The most frequently observed cross-links were located near the centre of the coiled-coils between K802 of SCM2 and K458 of SMC4, and nearby, between K396 of SMC4 and K869 of SMC2 (figure 2). Other linkages formed along the length of the SMC2–SMC4 coiled-coils, revealing that the SMC core of purified condensin I has a rod shape.

Cross-linking confirmed that the CAP-H kleisin subunit links the SMC2 and SMC4 heads, as well as forming a platform for the CAP-G and CAP-D2 subunits. The SMC2 head (K222) cross-linked within the amino-terminal half of CAP-H (K199), whereas the N-terminus of SMC4 was cross-linked towards the CAP-H C-terminus (K655). We did not detect cross-links between the N-terminal region of CAP-H and the coiled-coil of SMC2, analogous to those between Scc1 and SMC3 found in one recent study [53]. CAP-G was cross-linked to the middle part of CAP-H (amino acids 400–500), and CAP-D2 cross-linked near the CAP-H C-terminus (figure 2*a*). Together, these observations confirm atomic force microscopy data from the Yanagida laboratory [21], as well as a recent elegant cross-linking analysis of the non-SMC subunits of condensin by the Haering laboratory [34]. Thus, equivalent subunits in yeast and chicken condensin have similar arrangements.

Analysis of band ii, the least abundant of the cross-linked species, yielded 29 high-confidence linkage sites (figure 2*b*). All cross-links observed in band ii were also observed in band i.

Cross-linked condensin band iii provided the most comprehensive linkage map (110 high-confidence linkage sites), and included information about proximities between all the condensin subunits (figure 2*c*). A difference map made by subtracting the cross-links unique to band i from those found in band iii revealed that the bulk of the cross-links observed only in band iii were intermolecular (electronic supplementary material, figure S1a). Few new intramolecular cross-links were observed.

We identified multiple cross-links along the entire length of the coiled-coils. These included all the cross-links that we observed in bands i and ii plus a number of others linking SMC2 to SMC4. Detailed modelling of the condensin coils (see below) can account for 98% of observed SMC2–SMC4 cross-links, suggesting that they are probably formed within individual complexes.

The non-SMC proteins were cross-linked to the SMC head domains at the very base of the coiled-coils, but not to the hinge domains. Specifically, SMC2 was linked both to CAP-H and to CAP-D2. CAP-H was also linked to the SMC4 head (K133 and K281). CAP-D2 was cross-linked to the SMC4 coiled-coil and also to CAP-H at several points. CAP-H also formed several cross-links with CAP-D2.

To gain further information on the architecture of the coiled-coils, we analysed the SMC2/SMC4 complex on its own by performing a pull-down of SBP-tagged SMC2. Cross-linking of the purified SMC2/SMC4 complex yielded a single high molecular weight product in which only SMC2 and SMC4 peptides were detected by mass spectrometry (electronic supplementary material, figure S1b). This band was excised from the gel and analysed by mass spectrometry.

In the resulting linkage map (figure 2*d*), cross-links were particularly abundant along the coiled-coil regions, positioning the SMC2 and SMC4 coils relative to one another. These linkages indicate that the SMC2 and SMC4 coiled-coils can approach each other to within approximately 27 Å along their entire length. Furthermore, the linkages were consistently aligned across a folded depiction of the molecules, suggesting that the position of the coiled-coils relative to one another was highly reproducible (electronic supplementary material, figure S1c). Thus, the existence of multiple conformations or a high degree of flexibility of the complex in solution are unlikely.

The coiled-coils in the SMC2/SMC4 subcomplex were positioned in the same way as in the condensin holocomplex. Consistently, the same lysine residues were linked together, although more cross-links were detected. Although the globular domains were again involved in only very few cross-links, the observed links confirmed the proximity of the hinge domains of SMC2 and SMC4. The globular domains were found not cross-linked to the middle of the coiled-coils, but only to their ends.

The wealth of cross-linking data obtained in these experiments allowed us to create a three-dimensional structural model of the SMC2/SMC4 subcomplex over its full length that included the extensive coiled-coil structure (see §3.6).

### 3.3. Preliminary architecture of isolated cohesin complex

In parallel with the analysis of condensin, we also conducted a preliminary CLMS analysis of isolated cohesin complex. Cross-linking cohesin also yielded three high molecular weight products, each containing SMC1, SMC3, Rad21/Scc1 and STAG2/SA-2 (electronic supplementary material, figure S2a). The cohesin subunit arrangement deduced from cross-linking confirmed previous observations, with the head domains forming a platform for the non-SMC subunits [4,19,31,58]. The N-terminus of Rad21 was linked near the SMC3 head (electronic supplementary material, figure S2b). The SA-2 protein was also cross-linked to the head of SMC1. We did not detect linkages connecting SA-1 with the complex.

Similar to SMC2/SMC4, we observed multiple linkages connecting SMC1 with SMC3, indicating that the coiled-coils can approach each other along their entire lengths in purified cohesin (see also [53]). Those cross-links were not as well aligned as they were in condensin (electronic supplementary material, figure S2d). Occasionally, one lysine cross-linked to several others, forming linkages that would probably be mutually exclusive owing to distance constraints on the cross-links. Together, these observations suggest that the cohesin coils may be more flexible than their condensin counterparts. The ability of long coiled-coils in SMC proteins to adopt different structures has been discussed by others [9,18,20,21].

A tempting hypothesis for both cohesin and condensin is that the coiled-coils are close together when the complexes are not bound to chromosomes and open up to encircle the sister chromatids upon binding to DNA. We therefore attempted to analyse both complexes *in situ* by cross-linking in intact mitotic chromosomes.

### 3.4. Architecture of condensin *in situ* in mitotic chromosomes

**Figure 3.**
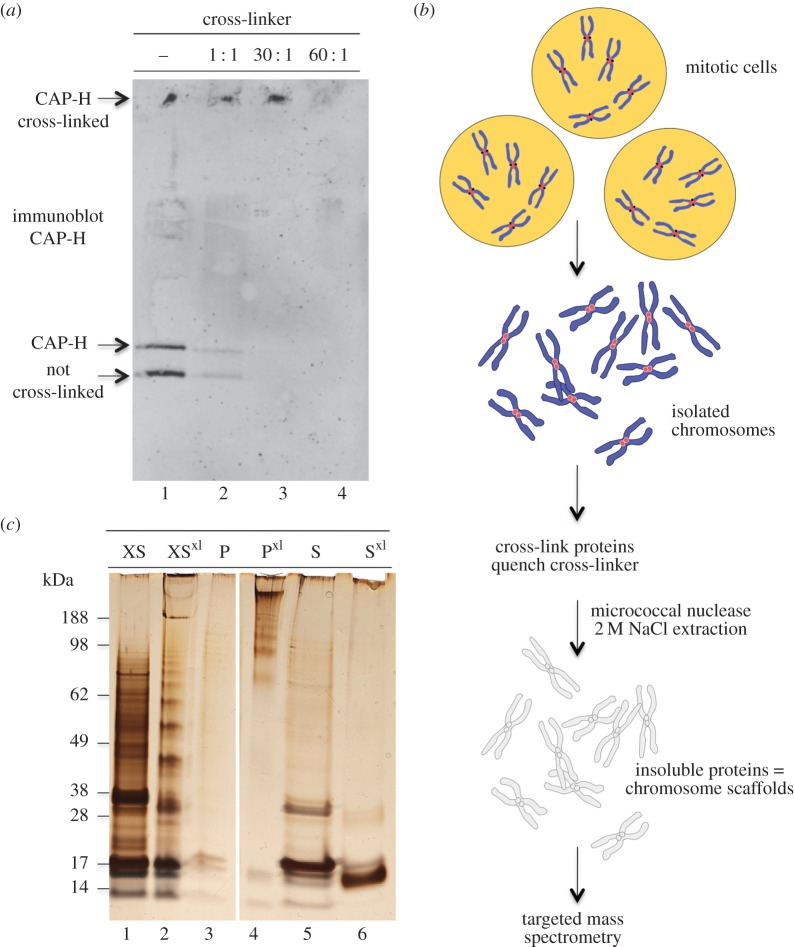
Cross-linking of condensin *in situ* in isolated mitotic chromosomes. (*a*) Immunoblot of the isolated chromosomes cross-linked with increasing amounts of BS3, probed using CAP-H antibodies. Purified non cross-linked condensin (lane 1) serves as control. (*b*) Protocol of sample preparation for cross-linking/targeted mass spectrometric analysis of condensin and cohesin on chromosome. (*c*) Chromosome scaffolds visualized by SDS–PAGE and silver staining: XS, isolated chromosomes; XS^xl^, cross-linked chromosomes; P, non-cross-linked pellet after scaffold extraction; P^xl^, cross-linked pellet; S, non-cross-linked supernatant; S^xl,^ cross-linked supernatant. The chromosome scaffold preparation step reduced the sample complexity from over 4000 to 610 proteins.

To establish the structure of active condensin and cohesin complexes *in situ*, we cross-linked intact isolated mitotic chromosomes [59]. Isolated chromosomes were incubated with increasing amounts of BS3 cross-linker to find suitable conditions for condensin cross-linking (figure 3*a*). The cross-linking behaviour of CAP-H was monitored by immunoblotting. A 30× weight excess of BS3 relative to the amount of total chromosomal protein was needed to efficiently cross-link CAP-H on chromosomes. With less cross-linker, non-cross-linked CAP-H was detected in SDS–PAGE. When more cross-linker was added, the CAP-H signal was lost—owing either to aggregation of complex or to modification of the epitope recognized by the antibody.

Isolated mitotic chromosomes contain over 4000 proteins [59]. This translates to a hugely increased number of peptides compared with what was observed with purified condensin, and is a background against which cross-linked peptides are less easily seen. Because the mass spectrometer acquires a constant number of spectra per unit time, when the overall number of peptides is greatly increased proportionally fewer of the cross-linked peptides will be detected.

In order to reduce the total peptide load in the mass spectrometer and increase the likelihood of detecting cross-linked peptides, the cross-linked chromosomes were digested with micrococcal nuclease and extracted with 2 M NaCl, yielding the chromosome scaffold fraction (figure 3*b*) [60]. This removed most of the very abundant histones and reduced the total number of proteins present to approximately 600. The scaffold fraction (figure 3*c*, lane 4) was then run in SDS–PAGE, and the area of the gel containing condensin (identified by immunoblotting for CAP-H) was excised and analysed by targeted mass spectrometry after strong cation exchange (SCX) chromatography to enrich for cross-linked peptides (Materials and methods). Mass spectrometry analysis used an inclusion list (electronic supplementary material, table S2) to focus the analysis on cross-linked peptides from condensin and cohesin identified in the previous *in vitro* studies. This decreased the time spent on analysis of other cross-links and linear peptides coming from the other proteins present in the scaffold fraction.

**Figure 4.**
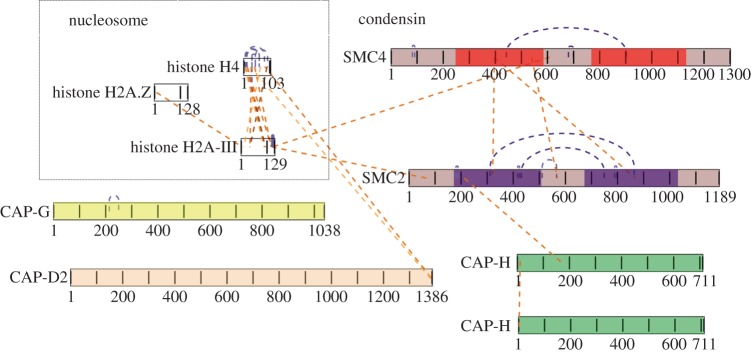
Condensin cross-links detected *in situ* in mitotic chromosomes. Linkage map of condensin complex cross-linked *in situ* in mitotic chromosomes visualized using xiNET (www.crosslinkviewer.org) [57]. Three linkages connect SMC2 with SMC4, two of them in the middle of the coiled-coils. One linkage connects the head of SMC2 with CAP-H. Nine intramolecular linkages provide information about the topology of SMC4 and SMC2 proteins. Four linkages indicate direct interactions between H2A or H4 and condensin.

In total, 14 cross-linked peptides were identified from condensin. These included nine intramolecular cross-linked peptides involving either SMC2 or SMC4, two cross-links between the SMC2 and SMC4 coiled-coils, one cross-link connecting the SMC2 hinge with a region close to the SMC4 hinge, one cross-link between K209 from SMC2 and CAP-H and one cross-link between the N-termini of two CAP-H proteins (figure 4). The intramolecular cross-links confirmed that the topology of coiled-coils and globular domains found for isolated condensin is conserved *in situ* in intact chromosomes. Strikingly, both cross-linked peptides that connect the SMC2 and SMC4 coiled-coils link the centre of the coils. These cross-links are of high confidence because they show almost full b- and y-ion series for both peptides (electronic supplementary material, figure S3a,b). Thus, the centres of SMC2 and SMC4 coiled-coils can closely approach one another when the condensin complex is assembled in chromosomes. Our data cannot distinguish whether the SMC2–SMC4 linkages form within a single condensin complex, or between two adjacent complexes. However, modelling of the condensin coils (see below) suggests that they can form within a single complex.

Unambiguous evidence for a close association of condensin complexes within chromosomes was provided by a high-confidence linkage between the N-terminal peptides of two different molecules of CAP-H (electronic supplementary material, figure S3c). The ability of condensin pentamers to form higher-order multimers was also supported by native PAGE of non-cross-linked condensin complex which formed a smear extending from 700 kDa to above the 1236 kDa marker (electronic supplementary material, figure S2b). A previous electron microscopy study showed that condensin accumulates in miniclusters at crossing points of the chromatin network [61].

For the less abundant cohesin complex, we observed only a single intramolecular cross-link between the head of SMC1 and SA-2 (electronic supplementary material, figure S3d). Interactions between the coiled-coils were not detected, possibly because the coils are separated by entrapped chromatin fibres. Interestingly, SA-2 was also cross-linked to the kinetochore protein CENP-M [62,63] and SMC1 was cross-linked to ataxia telangiectasia mutated (ATM), a serine/threonine protein kinase that is recruited and activated by DNA double-strand breaks [64,65]. Because those cross-links must be relatively abundant in order to be detected against the background of other peptides, the interactions are likely to be biologically significant.

The paucity of cross-links detected on whole chromosomes using targeted mass spectrometry reveals the present limitations of cross-linking proteomic technology when applied to complex protein mixtures. Further fractionation of the chromosome sample might allow observation of additional cross-links involving the SMC proteins. It may also be that this will only be achieved when selective enrichment of cross-linked peptides becomes possible.

### 3.5. H2A and H4 are reproducibly associated with condensin on mitotic chromosomes

Cross-linking analysis of isolated condensin revealed that H2A and H2A.Z are present in the pull-downs and interact with the SMC hinge domains via their N-terminal tails. Specifically, Ser20 of H2A was found linked to Lys754 of SMC4, whereas Lys5 of H2A.Z was linked to Thr698 of SMC2. Analysis of the peptide spectra allowed identification of these cross-linked species with high confidence (electronic supplementary material, figure S4).

In the *in situ* cross-linking analysis, we found peptides linking the condensin complex with both histones H2A and H4. The C-terminal tail of H2A (Lys119) was linked to the hinge domain of SMC4 and to the head domain of SMC2 (figure 4—note that cross-links observed only *in vitro* are not shown in this figure). This agrees with data published by the Watanabe laboratory [66] and reveals that both the hinges and the heads of SMC proteins bind to chromatin. The *in situ* cross-linked peptide spectra are shown in the electronic supplementary material, figure S5a,b and the position of these cross-links on the nucleosome is shown in the electronic supplementary material, figure S6 [67].

We also observed cross-links between H4 and the C-terminus (Thr1382) of CAP-D2. These cross-links involved both the N-terminal (Lys 32) and C-terminal tails (Thr 83) of H4 (figure 4 and electronic supplementary material, figure S5c,d). It was previously reported that H4 mono-methylated on K20 was involved in binding condensin II to chromosomes via interactions with the HEAT repeat subunits CAP-D3 and CAP-G2 [68].

Further support for the notion that H2A and H4 dock condensin to chromosomes is provided by the fact that these were the most abundant histones in the purified condensin pulldowns according to emPAI [69] (10 000 and 100-fold more abundant than H3, respectively). In addition, 2 M NaCl was apparently less efficient at extracting H2A and H4 from cross-linked chromosomes, whereas cross-linking did not prevent extraction of H2B (compare figure 3*c* lanes 5,6). This difference may reflect cross-linking of H2A to one or more of the scaffold proteins. BS3 does not efficiently cross-link the histone octamer (2010, unpublished data).

### 3.6. A ‘draft’ three-dimensional structure of the entire SMC2/SMC4 core of condensin

The condensin complex fulfils the prerequisites for computational assembly of a three-dimensional structural model. Crystal structures of several homologues of the human SMC head and hinge domains have been determined to atomic detail and served as templates for modelling these globular domains of SMC2 and SMC4. Additionally, the remarkable density of high-confidence cross-links we observed in the coiled-coil segments (figure 2*a–d*) allowed us to assemble a low-resolution model of the SMC2/SMC4 dimer over its full-length, in spite of the lack of a homologous template structure for the anti-parallel coiled-coil segments. This model combines five modelled fragments of the coiled-coil for each subunit with the homology-modelled heads and hinges in a three-dimensional arrangement that is compatible with the experimental data and consistent with the structural knowledge and methodology available to date. We provide the overall assembly here as a disjointed three-dimensional coordinate model (electronic supplementary material, data file S1) so it can be used by others, and with the cautionary note that our confidence in the atomic coordinates differs for different portions of the assembly.

**Figure 5.**
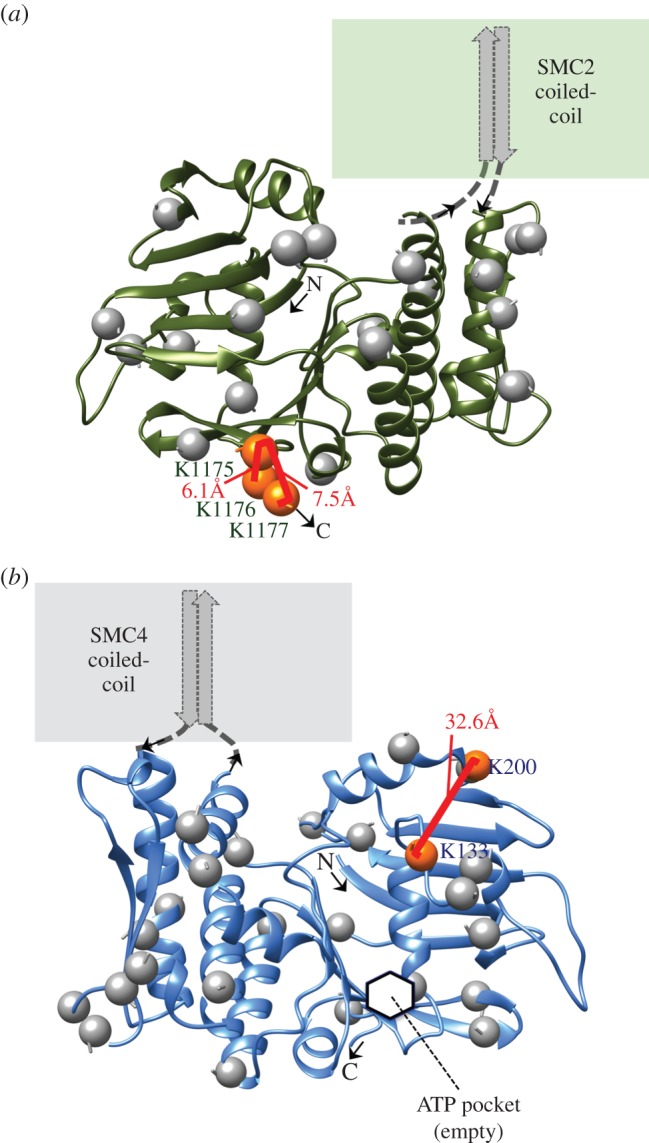
Homology models of SMC2 and SMC4 head domains. Ribbon diagrams of the bipartite head domains of chicken (*a*) SMC2 (residues M1–E167 and L1030–K1177) and (*b*) SMC4 (residues L79–E249 and L1129–A1280). Intradomain cross-links between lysines (orange spheres) are annotated with their Xwalk SAS distances [70]. Unlinked lysines are marked by grey spheres. The inferred location of the ATPase active site is pointed out on SMC4 (hidden in the view of SMC2). Images produced with UCSF Chimera v. 1.9.

We modelled the bipartite head (ATPase) domains (figure 5*a,b*) using as template the crystal structure of the homologous archaeal SMC from *Pyrococcus furiosus* co-crystallized with the kleisin subunit ScpA (PDB: 4I99 chain A) [71] and sharing 34% and 36% sequence identity to the modelled regions in our chicken SMC2 and SMC4, respectively. In bacteria and archaea, where homodimeric SMC protein complexes form, the closest homologues of the heterodimeric condensin component proteins SMC2 and SMC4 are also the closest homologues to the cohesin components SMC1 and SMC3 [72]. At the time of modelling, there was no crystal structure of a eukaryote condensin head domain. The models were built from target-template alignments on the archaeal SMC head domains, and their robustness confirmed by alternatively using those from an evolutionarily approximately equidistant bacterial SMC template from *Thermotoga maritima* (PDB: 1E69 Chain A) [73] (data not shown). Both template structures were crystallized without substrate (ATP). Thus, the modelled SMC2 and SMC4 head domain fragments should be regarded as three-dimensional representations of the molecular structure of the head regions in the apo-form of the ATPase.

**Figure 6.**
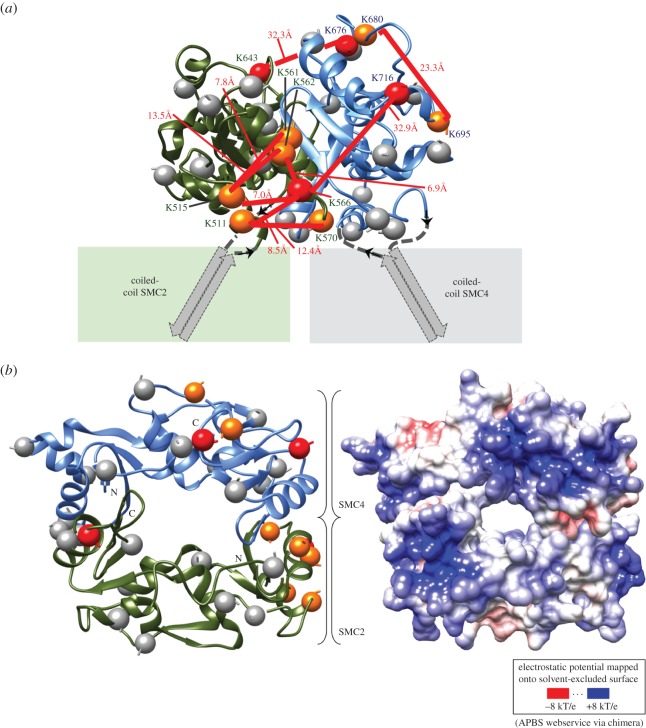
Homology models of the SMC2 and SMC4 hinge dimer. The modelled hinge fragments (SMC2 residues R507–A667; SMC4 residues S592–S762) viewed from the side are validated by nine cross-links (*a*), and the strongly basic surface electrostatics when viewed from the top corroborate the ability of this region to bind DNA (*b*). Colouring and annotation follows the scheme used in figure 5. In addition, lysines engaged in at least one intermolecular cross-link are shown as red spheres. Images and rendering with UCSF Chimera v. 1.9 interfacing with APBS [74].

For the hinge portion (figure 6), we chose the crystal structure of cohesin (SMC1/SMC3) from mouse (PDB: 2WD5 chains A and B) [17] as the most suitable template structure at 28% and 25% identity to chicken SMC2 and SMC4, respectively. The available structure of the murine condensin hinge at the time of modelling (PDB: 3L51, 68% and 71% identical to the modelled fragments) was also considered while building the model but the partially open conformation captured in that crystal had been deemed potentially unrealistic by its authors [15] and the closed ring-like arrangement observed in the cohesin structure was compatible with our cross-link data. A more recent modelling study of *Schizosaccharomyces pombe* condensin has suggested that opening of the ring-shaped hinge proximal to the sites of coiled-coil insertion may have a role in DNA binding, and that the opened hinge may be phosphorylated at sites that are normally hidden within the ring as a result of a novel activity of the condensin ATPase domains [75]. Visualization of the electrostatic properties of the hinge surface revealed a large basic patch (figure 6*b*), which is consistent with this region of the molecule binding to DNA [13–15].

No cross-links were used to produce the modelled three-dimensional structures of the SMC head and hinge domains. Thus, the 12 high-confidence cross-links within these regions (figures 5 and 6) allowed an independent experimental assessment of the predicted structures. Indeed, all solvent-accessible surface distances between cross-linked lysine Cβ-atoms (calculated by Xwalk [70]) were within the author-recommended threshold (less than 34 Å), averaging 16 ± 11 Å. As an important first result from our modelling, the homology-modelled head and hinge fragments allow us to refine the predicted boundaries between the segments in SMC2 and SMC4 that form the head, hinge and by implication coiled-coil regions (often referred to as d1–d5; table 1).

**Table 1.** Sequence- and structure-based boundary predictions for chicken SMC2 and SMC4.

	d1 head	d2 coil^a^	d3 hinge	d4 coil^a^	d5 head
SMC2^c^	1–167	168–506	507–674	675–1028	1029–1189
SMC4^d^	79–250	251–591	592–766	767–1128^b^	1129–1300

^a^Coiled-coil segments (maximum estimates).

^b^Includes a Pro-rich (not-coiled-coil) disruption 1035–1067.

^c^Sequence accession code IPI:IPI00579121.1.

^d^Sequence accession code IPI:IPI00573837.3, residues 1–78 are predicted to adopt disordered structure.

In contrast to the cross-link-independent steps yielding the head and hinge models, cross-links were essential for attempting to model the extensive regions of anti-parallel coiled-coil that comprise much of the SMC2/SMC4 dimer. In doing so, we did not presume that the coiled-coil segments are regular over their entire lengths, but rather let the cross-links provide the approximate relative spatial alignment of the two anti-parallel helix segments forming the coiled-coils.

**Figure 7.**
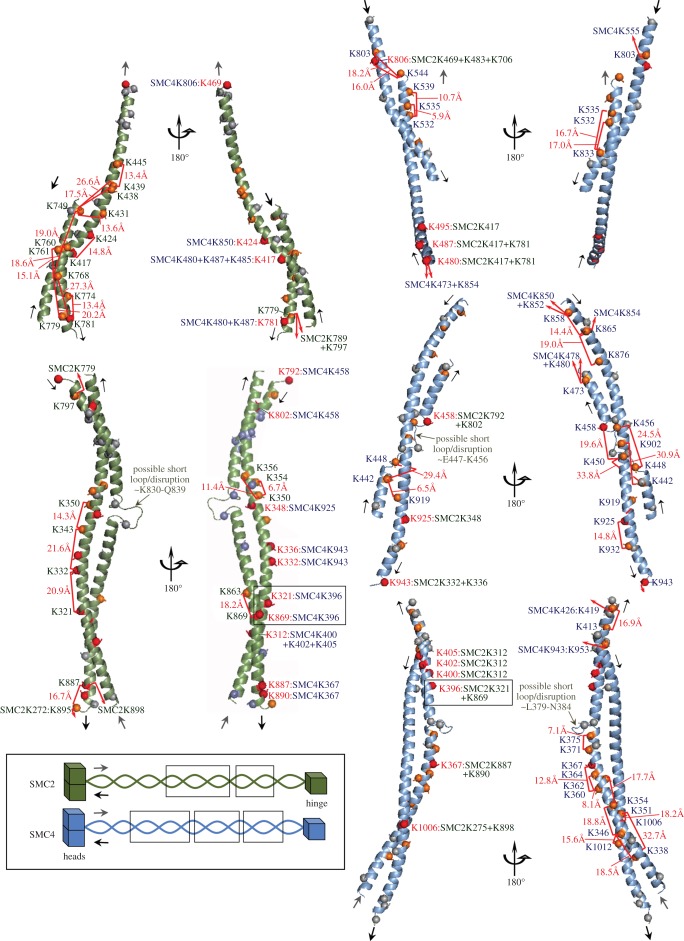
Some of the building blocks used to assemble the central portion of the condensin anti-parallel coiled-coils. Five of the 10 coiled-coil fragments modelled in this study are shown in two views each, providing full annotation detail of intra- and interdomain cross-links (red brackets with Xwalk SAS distances if both lysines are on the same fragment). Intermolecular cross-links are specified in the inner panel images from residues numbered in red font. These fragments span the central portion of the coiled-coil and include two sites with multiple intermolecular links (see also figure 8*c*). Their location in the three-dimensional model is shown schematically in the overview schematic (SMC2 residue ranges 395–469 + 746–786 (top), 293–386 + 792–895 (bottom); SMC4 residue ranges 479–544 + 793–845 (top), 431–477 + 855–945 (middle), 342–421 + 949–1034 (bottom). Images produced with PyMOL v. 1.7 (Schrödinger, LLC).

We accomplished this by identifying possible sites of irregularity (see Materials and methods) to break each segment into fragments, and then produced two-stranded anti-parallel coiled-coil models compatible with the cross-links by template-based modelling onto a crystal structure of Beclin-1 from rat (PDB: 3Q8T) [76]. Beclin-1 is neither a homologue nor a nuclear protein, but its coiled-coil region was the longest anti-parallel two-helical coiled-coil resolved to atomic detail at the time of writing that conforms well to the canonical pair-wise geometry and sequence (13 heptad repeats and approx. 127 Å pitch) [76]. Compatibility of the 10 coiled-coil fragments with all 16 interdomain cross-links within them was confirmed by the Xwalk solvent-accessible surface (SAS) criterion (less than 34 Å; average Cβ–Cβ SAS distance 18 ± 5.7 Å). Most of these cross-links and the central fragments are illustrated in figure 7.

**Figure 8.**
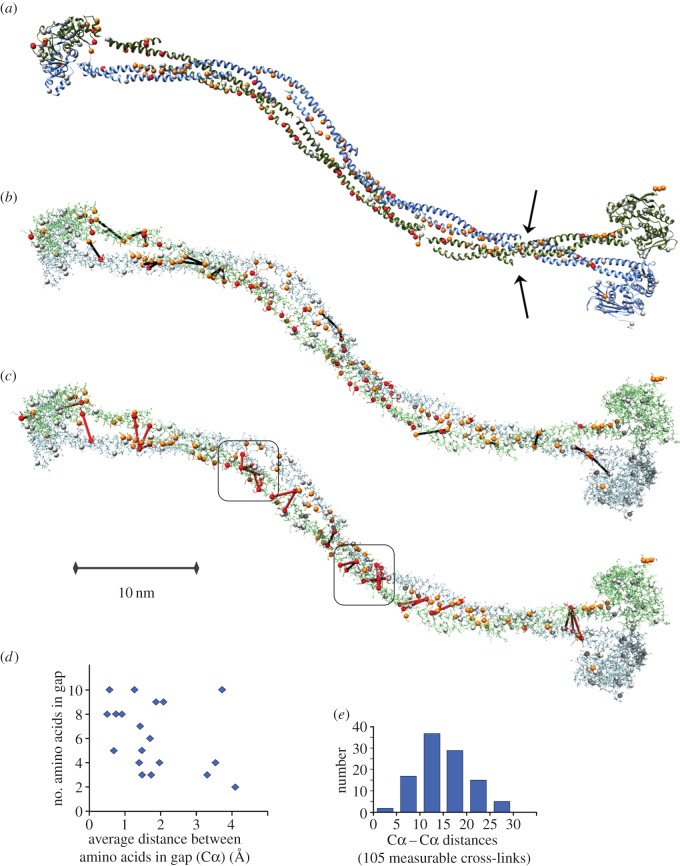
Low-resolution approximation of the three-dimensional structure of the SMC2/SMC4 core of chicken condensin generated through template-assisted rigid assembly of 13 fragments. (*a*) Ribbon depiction of the 1096 SMC2 residues (92%) and 1111 SMC4 residues (85%) included in the model. Orange and red spheres depict Lys–Cα found in at least one high-confidence cross-link (grey spheres are unlinked lysines). Arrows mark where four sites on SMC2 and SMC4 predicted as possibly irregular in 2002 (loops I and III according to Beasley *et al*. [43]) line up on the modelled dimer although helical fragments were assembled solely based on the cross-linking data. (*b*) All-atom depiction of the model. Black lines denote the intramolecular links found between ‘domains' (table 1), which includes those between the anti-parallel helices in the coiled-coils that we used to derive/confirm their approximate relative alignments in each modelled fragment. The Cα–Cα distance average across these interdomain intramolecular cross-links (nine in SMC2; 12 in SMC4) was 16 ± 5.9 Å. The X-walk SAS Cβ-distance average over the 16 in-fragment cross-links among them was 18 ± 5.7 Å. For comparison, the Cα–Cα distance average of the 57 intradomain cross-links (not shown) was 12 ± 4.6 Å and the X-walk SAS Cβ-distance average over the 53 in-fragment cross-links among them was 16 ± 7.3 Å. (*c*) Red lines depict the 27 intermolecular lysine cross-links easily accommodated in this individual SMC2/SMC4 dimer (three links were rejected as not compatible). These cross-links suggest a close proximity of the two coiled-coils in the rod-like conformation of the heterodimer. The Cα–Cα distance average for these intermolecular cross-links was 21 ± 4.3 Å. Boxes enclose two clusters of intermolecular cross-links that are best modelled as a quadruple-stranded coil. (*d*) Fit of the assembled model to the spatial junction constraint between modelled fragments (see Results). Average distances per residue are shown for 19 junctions where between two and 10 residues were omitted in the modelling in between fragments, and constraints were imposed. For reference, typical distances for residues in α-helical and β-strand conformations are 1.5 and 3.4 Å, respectively. (*e*) Histogram of all measurable Cα distances in the model between cross-linked lysines, including the linkages shown in panels *b* and *c* and the 57 intradomain linkages. Molecular graphics produced with UCSF Chimera v. 1.9.

Finally, we assembled the modelled coiled-coil fragments to form a ‘three-dimensional draft’ of the full-length SMC2/SMC4 heterodimer (figure 8). Here, we sought a solution that would be compatible with as many of the mapped intermolecular cross-links as possible. There is currently no automated method capable of assembling an elongated structure such as this. Thus, we began to model two locations where multiple cross-links positioned SMC2 and SMC4 in close proximity (boxed in figure 8*c*) by locally copying the interhelical angles from the classic ‘dimer of coiled-coil’ bundle of the repressor of primer (Rop) protein structure (PDB: 1ROP from *Escherichia coli*) [77]. Next, we added the remaining fragments including the head and hinge domains, and manually assembled the whole into a ‘disjointed’ three-dimensional model in which we respected three primary structural constraints: (i) continual left-handed winding of the anti-parallel coiled-coil helices around one another, (ii) spatial distances between the fragments (we refer to this as a ‘junction criterion’) commensurate with the number of resides not modelled and including a 1–2 Å intentional additional off-set to emphasize and counteract the limitations of coiled-coil modelling and rigid fragment assembly (figure 8*d*), (iii) Cα distances between lysines found in intermolecular cross-links in our experiment less than 30 Å (again we added some tolerance to the empirical/experimentally determined value of 27.4 Å [51], to account for modelling uncertainty). The distribution of Cα–Cα distances for 105 measurable cross-links is shown in figure 8*e*.

The resulting ‘draft’ model visualizes the approximate locations of 1096 residues (92%) of SMC2 and 1111 residues (85%) of SMC4, in the SMC2/SMC4 core complex captured in our cross-linking experiments (figure 8). Its atomic coordinates as well as rendering scripts for the two commonly used molecular visualization programs PyMOL (Schrödinger LLC, http://www.pymol.org) and UCSF Chimera [78] (http://www.cgl.ucsf.edu/chimera) are provided in the electronic supplementary material, data file S1, to facilitate use of the model by other laboratories.

This model stems from an experimental–computational hybrid approach, with cross-link information contributing vitally (except in the homology-modelled head and hinge domains). By contrast, a purely computational attempt would probably have failed owing to irresolvable uncertainty in the alignment of the two anti-parallel helices to one another in each coiled-coil fragment.

Altogether, our three-dimensional assembly explicitly accommodates 57 intradomain cross-links (33 in SMC2, 24 in SMC4), 21 interdomain intramolecular cross-links (9 in SMC2, 12 in SMC4) and 27 intermolecular cross-links. An additional nine cross-links appeared to be implicitly compatible although only one partnering lysine was included in the model for eight of these links, and neither lysine was modelled for the ninth link (where only four residues separate them in sequence).

Out of 120 high-confidence cross-links in total, we deemed only three intermolecular links to be incompatible, i.e. we could not accommodate them simultaneously with the others even by allowing a domain–domain rotation between the coiled-coil and globular domains that deviated from the currently available template structures. These cross-links could possibly have arisen from contacts between adjacent condensin pentamers.

## 4. Discussion

We have combined classic molecular modelling with a novel cross-link-constrained modelling strategy tailored to long coiled-coils to produce a draft structure of the SMC2/SMC4 dimer from chicken condensin. The extensive anti-parallel coiled-coils of SMC2 and SMC4 were excellent substrates for the lysine-directed cross-linker BS3, and 85/120 high-confidence cross-links mapped within these regions. The head and hinge domains acquired many fewer cross-links, but we could confirm that the N-terminus of the CAP-H kleisin binds the SMC2 head whereas its C-terminus associates with the SMC4 head. We did not, however, find evidence for the CAP-H N-terminus intimately associating with the SMC2 coiled-coil, as seen for analogous components in bacterial condensin [71] and in cohesin [32,53].

The principal surprise from our study was that the coiled-coil domains of SMC2 and SMC4 are closely apposed along their entire lengths. This was not expected, given the elegant and convincing studies showing that yeast condensin associates with chromatin as a topological ring similar to what has been proposed for cohesin [23,79]. We postulate that when not actively engaged on mitotic chromosomes, condensin adopts a closed structure similar to that observed by electron and atomic force microscopy [18,20,21].

Given the early success in deducing their presence from bioinformatics analysis, one might imagine that it would be straightforward to predict the three-dimensional structures of coiled-coils from their amino acid sequence. However, predictions of heterodimeric coiled-coils are extremely challenging. This is because there is generally insufficient information in the amino acid sequences to accurately predict the spatial alignment of the two helical segments forming the coiled-coil with respect to one another. Sliding one helix forward or backwards by one heptad repeat of seven amino acids (roughly 10.5 Å) will frequently yield a coiled-coil of comparable stability and validity, from a purely structural point of view. A second problem is that with few exceptions, long coiled-coil regions adhere only approximately to the canonical geometry and 3.5 residue periodicity that results from supercoiling of two α-helices with average/idealized 5.0 Å radius and approximately 140 Å pitch [80,81]. When coiled-coil periodicity is disrupted by skips, stutters and stammers [82], this can often be accommodated without dramatically disrupting the supercoiling [41,83], but regular geometry is often disturbed by loops inserted between helical segments. Such irregularities can be crucial to the functions of coiled-coil proteins by offering binding sites for other proteins, as for the kinetochore protein NDC80 [58,84,85]. Interestingly, existence of the loop in the NDC80 coiled-coil was first demonstrated by CLMS [47]. There are no simple algorithms for precisely predicting such interruptions and very limited reference data on which they could be validated. Although evolutionary sequence analysis between close homologues is useful for discerning potential breaks by helping to define the heptad pattern (see Materials and methods), the conservation of structural detail may not extend to very distant homologues as it does in most globular domains. Altogether, this means that the majority of helpful and varied constraints for prediction and modelling of globular protein three-dimensional structures and complexes are lacking, or ill-defined, when the targets are long heterodimeric coiled-coils.

**Figure 9.**
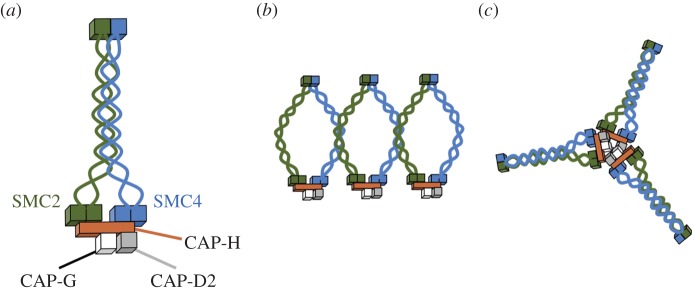
Possible models of condensin complex structure based on cross-linking data. (*a*) Diagram of condensin as a rod-shaped complex suggested by the cross-linking data. (*b*) Alternative model suggesting that on chromosomes, cross-links between the SMC2 and SMC4 coiled-coils could arise owing to side-by-side association of condensin holocomplexes. (*c*) Cross-linking suggests that condensin can form multrimers (possibly trimers *in vitro*) where CAP-H proteins interact.

Although crystal structures of several homologues of the human SMC head and hinge domains have been determined to atomic detail and served as templates for modelling the globular portions of SMC2 and SMC4, assembly of a draft structural model for SMC2/SMC4 was only made possible here by inclusion of constraints from the cross-linking analysis. This enabled us to pursue a template and fragment based approach and assemble the 13 fragments that were compatible with 117/120 high-confidence cross-links derived from the various condensin preparations into a low-resolution three-dimensional view of the entire SMC2/SMC4 dimer. The model reveals an intimate rod-like arrangement of the SMC2/SMC4 molecules dictated by the numerous, regularly arranged, intermolecular cross-links in the coiled-coil regions. Intriguingly, the remarkable consistency of the cross-link data with a single model seems to indicate that a single rod-like form [18] predominated in our samples (figure 9*a*), although alternative, V-shape conformations would not be detected with this protocol. Although it is not meaningful to talk of ‘resolution’ in a model structure such as ours, constraints owing to the presence of multiple cross-links and amino acid spacing in junctions between modelled fragments mean that the coiled-coil register of our model is likely to be correct within one heptad repeat (see Materials and methods).

In addition to the precise domain boundaries and structural parameters derived from this analysis, three noteworthy observations arise from considerations relating to the model.

First, the cross-linked SMC2/SMC4 complex appears to exhibit structural flexibility (and/or the ability for controlled movement) at the connection points between the long coiled-coil and globular domains with regard to the angle between coiled-coil and head and hinge domains.

Second, two previously proposed disruptions to sequence periodicity within the long coiled-coil, referred to as ‘loop I’ and ‘loop III’ [43], line up opposite one another in our three-dimensional model. That is, not only do loop I and loop III, which are at opposite ends of the coiled-coil in both SMC2 and SMC4, line up opposite one another in the anti-parallel coiled-coil within each molecule, the two loops from SMC2 also line up opposite their counterparts from SMC4 in the four-stranded coil. Our analysis additionally defines a proline-rich 33 residue insertion within SMC4 (‘loop III’ residues 1035–1067, not modelled).

A recent alternative cross-linking approach has described the overall geometry of the bacterial condensin MukB [82] and revealed the presence of multiple interruptions of its coiled-coil. These interruptions, termed ‘knuckles', have been suggested to impart flexibility to the coiled-coils in MukB and bacterial SMC [37]. Further experiments are required to determine the functional significance of the analogous structural features in condensin.

Third, because SMC coiled-coils are flexible [18,20,37], we cannot exclude the possibility that native purified condensin is a ring that becomes progressively ‘zipped together’ by the cross-linker. We suspect that this is unlikely, however, as the cross-links between SMC2 and SMC4 coiled-coil domains are highly regular and reproducible, suggestive of a unique packing of SMC4 against SMC2. This contrasts with the pattern of cross-links seen between SMC1 and SMC3 in isolated cohesin, where it appears that the coils can be trapped in a number of different states by the cross-linker. We note that similar cross-links were also seen in another recent study of cohesin [53].

Further support for the intimate association of the SMC2 and SMC4 coiled-coils comes from analysis of two regions in which we find multiple intermolecular cross-links (6 and 5, respectively, within two 26 residue windows), shown in figure 8*c*. Modelling these regions in three-dimensions could only be accomplished by locally inferring a four-helix bundle as shown in our model. While some conformational impact of cross-linking cannot be ruled out, we would intuitively not expect to find more than one such tightly cross-linked region had the rod-like structure been formed through cross-link-induced aggregation.

Given the convincing evidence that budding yeast condensin forms topological links around chromatin [24], we also attempted to see whether we could cross-link the coiled-coils of functional condensin in mitotic chromosomes. If condensin embraces chromatin fibres as proposed for cohesin [19,24], then cross-links between the SMC2 and SMC4 coils should not be observed. Cross-linking intact chromosomes and extraction of approximately 95% of chromosomal protein prior to mass spectrometry analysis [86] enabled us to detect a number of cross-links from functional condensin *in situ*. Importantly, we did observe two cross-links between SMC2/SMC4 near the exact centre of the coiled-coils. These cross-links reflect intermolecular contacts in our draft model of the isolated SMC2/SMC4 dimer. This could suggest that at least some of the condensin on mitotic chromosomes does have closely paired SMC2/SMC4 (i.e. is not encircling the chromatin fibre). It is possible that this reflects chromosome-associated condensin that is yet to be functionally activated.

It is also theoretically possible that these SMC2/SMC4 cross-links formed in trans between two adjacent condensin complexes (figure 9*b*). Condensin has been shown to bind chromatin in clusters, and our *in situ* analysis detected an interaction between the CAP-H N-termini, strongly suggesting that condensin complexes do associate closely with one another in chromosomes (figure 9*c*). However, detailed consideration of our model strongly suggests that the cross-links between the SMC2 and SMC4 coiled-coils are likely to be from within the individual complexes, and that at least the linked lysines in the middle of the coils may remain proximal also in active condensin.

Our data also confirm previous reports that the SMC hinge and head domains are involved in the docking of condensin to chromatin. We observed cross-links between histone H2A and the head domain of SMC2 and hinge of SMC4. These contacts are mapped onto the surface of a nucleosome in the electronic supplementary material, figure S6 [67]. We also detected cross-links between both the N- and C-terminal regions of histone H4 and CAP-D2. It had previously been reported that H2A is a receptor for condensin on chromosomes [66] and that H4 mono-methylated on K20 binds CAP-D3 of condensin II [68]. Our data confirm the association of CAP-D2 with histone H4, but suggest that K20 methylation may not be required for this association in mitotic chromosomes.

## 5. Perspectives

Recent crystal structures and cross-linking analysis are providing a wealth of structural information about eukaryotic SMC protein complexes. The initial low-resolution structure presented here, together with other recently published work, should enable a new era of precise structure-based mutagenic analysis of the condensin complex.

## 6. Material and methods

### 6.1. Purification of SMC2/SMC4 subcomplex, condensin holocomplex and cohesin complex

SMC2 knockout cells expressing SBP-tagged SMC2, CAP-H knockout cells expressing SBP-CAP-H and Scc1 knockout cells expressing 9Myc-tagged Scc1 [29,55,87] were grown as described previously [88] in 200 ng ml^−1^ of doxycycline for at least 48 h. When cells reached a density of 10^6^ per ml, nocodazole was added for a further 13 h to obtain a mitotic index of more than 80%. Cells were lysed in lysis buffer (50 mM HEPES, pH 7.5, 0.25 M NaCl, 0.5% NP-40, 30 μg ml^−1^ RNase A), supplemented with the protease inhibitors 1 mM PMSF (Sigma-Aldrich) and 1 µg ml^−1^ CLAP (chymostatin, leupeptin, antipain, pepstatin A; Sigma-Aldrich) for 45 min on ice. After sonication, cellular debris was removed by centrifugation at 20 000*g* for 10 min at 4°C. Cell lysates (4 × 10^8^) were incubated either with 300 μl of streptavidin–sepharose beads (Streptavidin Plus UltraLink Resin, Pierce) for 2 h at 4°C (SMC2/SMC4 and condensin) or with 200 μl of anti-c-Myc tag gel (MBL) in a column for 1 h at 4°C (cohesin) in a final volume of 10 ml on a rotary wheel. Beads were washed three times with wash buffer (50 mM HEPES, 0.5% NP-40, 0.25 M NaCl) on a rotary wheel for 5 min at 4°C and the proteins were eluted either twice in 600 μl of wash buffer containing 4 mM biotin (SMC2/SMC4 and condensin) or five times with 200 μl of c-Myc tag peptide (0.1 mg ml^−1^) in wash buffer (cohesin) on a rotary wheel for 30 min at 4°C. The eluents were analysed by SDS–PAGE and by immunoblotting.

### 6.2. Cross-linking of SMC2/SMC4, condensin and cohesin complexes

The mixing ratio of BS3 to complexes (SMC2/SMC4, condensin, cohesin) was determined by using 1 µg protein aliquots and a 30-, 90-, 270-, 810- or 5-, 15-, 30-, 60-, 120- or 3-, 30-, 90-fold weight excess (respectively) of BS3 cross-linker (Thermo Scientific) resuspended in DMSO at 300 µg µl^−1^. After 2 h, the reaction was quenched by addition of ABC to 50 mM for 30 min. The products of cross-linking were separated on a NuPAGE 4–12% bis–tris gel (Invitrogen) using MES running buffer and were Coomassie- or silver-stained.

Either 36 μg of purified SMC2/SMC4 or 100 μg of condensin complex, at 0.05 µg µl^−1^ in 50 mM HEPES buffer, 250 mM NaCl, 0.5% NP-40, 4 mM biotin, was cross-linked with 30-fold weight excess of BS3 for 2 h on ice. After 30 min quenching, the cross-linked complexes were separated in 4–12% bis–tris gel (Invitrogen). Also 100 μg of cohesin complex at 0.02 µg µl^−1^ was cross-linked in the same way.

### 6.3. Native electrophoresis of the condensin complex

Freshly purified condensin and cohesin complexes were separated in native PAGE Novex 3–12% bis–tris gels according to the manufacturer's instructions (Life Technologies).

### 6.4. Cross-linking of SMC complexes on chromosomes and scaffold preparation

Chicken chromosomes were purified as described previously [59]. To find the optimal ratio of protein : cross-linker, 1 μg of chromosomal protein was incubated with a 1-, 30-, 60-, 90-fold weight excess of BS3. The cross-linked proteins were analysed by immunoblotting. Anti-CAP-H antibody was used to reveal the ratio of cross-linker needed to optimally cross-link the condensin complex *in situ*.

Purification of chromosomes from 500 ml of DT40 wild-type culture was carried out 11 times, and each time the chromosomes were cross-linked with a 30-fold weight excess of BS3 for 2 h on ice, followed by quenching with 50 mM ABC for 30 min. The cross-linked chromosome samples were supplemented with 2 mM CaCl_2_, treated with micrococcal nuclease (40 μg μl^−1^; Worthington) for 30 min on ice, then diluted with an equal volume of freshly made TEE buffer (10 mM triethanolamine : HCl pH 9, 1 mM NaEDTA pH 9). Immediately, an equal volume of 2× NaCl lysis mix (20 mM Tris : HCl pH 9, 20 mM NaEDTA pH 9, 0.2% AMX, 4 M NaCl) was added. The samples were spun down at 14 000 r.p.m. for 5 min at 4°C. The pellet containing the scaffold proteins was re-suspended in 1 × SDS sample buffer, boiled for 5 min, sonicated for 15 min and boiled again for 5 min. The scaffold proteins were loaded into 20 wells of two 4–12% bis–tris gels (Invitrogen) and separated in MOPS buffer for 2 h. The very top area of each lane containing the condensin complex was cut out and in-gel digested. The extracted peptides were analysed by SCX-HPLC as previously described [51].

### 6.5. Sample preparation for mass spectrometry analysis

Bands containing the cross-linked complexes were excised from gels and in-gel digested following standard protocols. The cross-linked peptides were extracted from gel slices, acidified to pH 3.0 with 0.5% acetic acid and fractionated using the SCX-StageTip [51]. High salt fractions were diluted four-fold with 0.1% TFA and desalted using C18-StageTips [89] before MS analysis.

### 6.6. Mass spectrometry

Cross-linked peptides were analysed on LTQ-Orbitrap Velos (Thermo Scientific) on a 180 min or 240 min gradient, using CID collision energy at 35% and fragmenting the eight most intense peptide precursor ions with charge stages *z* = 3 or higher, per cycle. MS spectra were recorded at 100 000 resolution, and MS/MS spectra at 7500 resolution, both in the Orbitrap. When analysing scaffold samples, an inclusion list stating the *m*/*z* values of condensin and cohesin cross-linked peptides identified in the *in vitro* study was used to dictate the MS/MS analysis. First, the ions from the inclusion list were fragmented, and only if these were not detected were other peptides of *z* > 2 fragmented using dynamic exclusion.

### 6.7. Database searching

The MS/MS spectra peak lists were generated from the raw data files using the Quant module of MaxQuant v. 1.0.11.2 [90] at default parameters, except for choosing 200 as ‘top MS/MS peaks per 100 Da’. Cross-linked peptide spectra were searched using the software package Xi (ERI, Edinburgh) against *Gallus gallus* condensin and cohesin sequences uploaded from SwissProt or from the chicken IPI database (v. 3.49) modified as described for analysis of chicken mitotic chromosomal proteins [59]. Search parameters: MS tolerance 6 ppm, MS/MS tolerance 20 ppm, fixed modification carbamidomethyl on cysteine, variable modifications: oxidation (Met), DST/BS3-OH (Lys), DST/BS3-NH2 (Lys), the ‘Max. missed cleavages' was set to 4. Matched spectra and cross-linked peptide candidates were returned by Xi in pairs. The highest scored matched spectra were validated manually, and to each spectral match a confidence was allocated. A high-confidence match indicates that for the longer peptide almost all and for shorter peptides a minimum of three fragments were matched and all major peaks in the spectrum were accounted for. A low-confidence match indicates that one peptide matched essentially all observed fragments and a second peptide had up to three fragments matched with most of peaks in spectrum explained. Reverse peptide sequences were used as a decoy search. All matches had to be highest ranking and unambiguous in the target and decoy search.

### 6.8. Homology models for SMC head and hinge domains

Alignments between the head segments of SMC2 and SMC4 and template structures for modelling were obtained initially by online submission of the individual sequences to HHpred (http://toolkit.tuebingen.mpg.de/hhpred) [91], allowing up to three iterations of HHblits before comparison against pdb70. Minor manual adjustments in insertion/deletion regions were made to the alignment suggested by HHpred (ranked first by the server in January 2015 when searching with the SMC2 head fragments as modelled, with HHpred score = 285.15 at *E*-value = 4.7 × 10^−38^; ranked second for the SMC4 head fragments with HHpred score = 304.61 at *E*-value = 3.1 × 10^−40^) to ensure that they coincided optimally with loop structure in the head domain segments of template structure PDB: 4I99_A. Hinge region alignments with dimeric template PDB: 2WD5 were obtained the same way (in January 2015, the SMC2 hinge fragment ranked seventh, HHpred score = 154.81, *E*-value = 1.3 × 10^−23^; the SMC4 hinge ranked fifth, HHpred score = 165.11, *E*-value = 2.0 × 10^−25^). Atomic coordinates built on these target-template alignments were generated using modeller v. 9.2 [92], choosing the best out of 20 models based on objective function score and visual inspection. No cross-link data were used. The target-template alignments and the compatibility between the predicted secondary structure for the targets with those for the templates implicitly also redefined the boundaries between the head, coiled-coil and hinge domain segments (table 1).

### 6.9. Validation of cross-link data on SMC domain models

SAS distances between Cβ-atoms of cross-linked lysines were calculated from each modelled structure fragment using the Xwalk web server v. 0.6 [70] (http://www.xwalk.org), in validation mode. Thresholds for calculation were set to 40 Å to ensure proper calculation but all reported SAS distances were within the developer-suggested 34 Å cut-off. Where Euclidian distances are discussed in the text these refer to Cα–Cα distances measured in UCSF Chimera [78] on the atomic Cartesian coordinate set for the model as provided with this publication (electronic supplementary material).

### 6.10. Coiled-coil fragment models

Multiple sequence alignments of the newly defined coiled-coil segments (d2 and d4 in table 1) with close homologues were obtained by submission to the COILS/PCOILS server within the Bioinformatics Toolkit Tübingen suite [91] in December 2013, with PSI-BLAST enabled and default settings retained otherwise. For identifying potential ‘break-points' for fragmenting the model, these automatically obtained coiled-coil predictions and heptad periodicity position assignments by COILS/PCOILS were also considered; however, they were supplemented (and often overruled) with manual multiple sequence analysis heuristics, and with secondary structure predictions obtained via the Genesilico metaserver [93] (http://www.genesilico.pl/meta2). (There are various structurally documented examples of disrupted heptad periodicity in nonetheless regular coiled-coil segments in known structures, and conversely structural disruptions that are not easily correlated to disruptions in heptad periodicity). Specifically, we assigned potential break-points where at least one, and usually several, signs of aperiodicity were noted in the sequence alignment: (i) five or more consecutive alignment positions in which highly polar amino acids dominated; (ii) disrupted helix predictions according to three or more secondary structure prediction methods from different research groups; (iii) four or more consecutive positions that featured only hydrophobic amino acids; (iv) secondary structure ‘parsing’ gaps or amino acids [94] present in more than one third of the homologues; (v) strong indications of disruption of the sequence repeat pattern (not spanning multiples of seven positions) as revealed through alignment analyses with HHrep, HHrepID, and/or the COILS/PCOILS outputs at the Bioinformatics Toolkit site [91] corroborated by other heuristics. Ideally, we would like the fragment boundaries to coincide with locations where the coiled-coil structure features substantive disruptions, e.g. inserted loops. Owing to the scarcity of reference structures, there are no tested methods for identifying such locations. Our heuristics reflect a common-sense procedure to this end, without claiming that all irregularities can be identified this way or that all breaks in the model will precisely match a coiled-coil disruption. Accordingly, alternatives to the initially selected break-points were considered throughout the model assembly process. We also note that, at the limited resolution the model delivers, potential mispredictions in this prediction step would not significantly impact upon it, thanks to the inbuilt tolerance in the open junctions in between the modelled fragments (described in Results). Next, paired coiled-coil models were produced using modeller v. 9.2 (used as above) for the independently partitioned fragments of the two coiled-coil segments in each molecule, using d2–d4 interdomain cross-links for guidance and the crystallographically determined anti-parallel coiled-coil from Beclin-1 as modelling template (PDB: 3Q8T; 94 resolved residues in chain A, 95 in chain B, corresponding to BECN1_RAT residues 172–265 and 170–264 in Uniprot). At least two compatible target-template alignments for each segment were produced (typically off-set by seven positions reflecting realistic ranges of cross-link reach), differing in end-overhang of one of the helices and/or local disruptions if applicable.

Although the length of the BS3 cross-linker (27 Å) is significant relative to the length of one heptad repeat (approx. 10.5 Å), when multiple cross-links exist between helices, the register between paired helices in the coiled-coil becomes more constrained. Reflecting the uncertainty in each fragment, we produced alternative models by modifying the target-template alignments accordingly (by shifting by seven positions and/or considering alternative local disruptions). Of these alternatives we chose those models that were compatible with the Xwalk distance threshold and structurally realistic to be considered closely in the assembly. Altogether, we considered 23 modelled fragment structures (11 for SMC2, 12 for SMC4) more closely in this subsequent step, i.e. at least two alternatives for each fragment. Additionally, we confirmed in test simulations on the assembled model of the entire coiled-coil segment in SMC2 that we could fit alternative solutions to the coiled-coil regions and that shifting the register by around seven residues broke only small numbers (less than 20%) of cross-links. However, larger shifts of around 14 residues (approx. 21 Å) are generally incompatible, i.e. break large proportions of the cross-links.

### 6.11. Assembly of the SMC2/SMC4 ‘three-dimensional-draft’ structure

Selection and assembly of the fragments into a coherent three-dimensional representation of the molecule was accomplished largely manually, with help of the UCSF Chimera modelling environment [78], in observation of the criteria stated in the Results section and in consideration of a small number of additional intercross-links between the fragments. In this step, multiple models for each coiled-coil fragment were tried (see above), and boundary signals deemed strongest in sequence analysis were used initially. We also considered alternative boundaries also found by the heuristics if junction criteria (specified in Results, see also figure 8*d*) were difficult to fulfil. Owing to the small number of amino acids in most junctions (of the 24 junctions in the heterodimer, only two spanned more than 10 amino acid residues), imposing a maximum distance per residue for the gaps contributed strongly to the assembly by informing the choice between alternative fragments with differing coiled-coil register. We allowed exceptions to the junction criterion only for the gaps bracketing the closely packed four-helix arrangement built to accommodate the richest cluster of intermolecular cross-links, in order to accommodate all links in this region. Here, two of four junctions fit the criteria (and are included in figure 8*d*).

After assembling the fragments, unnaturally close contacts with side-chains were detected using Chimera's analysis functions for detecting clashes, and resolved following their rotamer selection/replacement protocol using the Dunbrack library [95], avoiding very rare rotamers (less than 1% probability). Within the electronic supplementary material to this paper, we provide atomic coordinates and rendering of the final model (PDB, Chimera and PyMOL formats).

## 7. Note added in proof

As this manuscript was being submitted, a structural study was published that described the hinge and adjoining coiled-coil regions in selected SMC protein complexes from *Bacillus subtilis*, *Pyrococcus furiosus* and *Saccharomyces cerevisiae* [96]. The results of that study are in close agreement with the model structure proposed here. Thus, the close proximity of the coiled-coils along their lengths, the requirement for steep hinge-to-coil angles deriving from this juxtaposition and the electropositive surface potential at the top of the closed hinge ring appear to be conserved from chicken to yeast and possibly beyond.

## Supplementary Material

SUPPLEMENTARY MATERIALS LEGENDS.docx

## Supplementary Material

Barysz et al. Supplementary PDFs

## Supplementary Material

Barysz Supplementary Table 2 - Inclusion List

## Supplementary Material

Barysz_et_al_Supplementary Data File 1

## Data accessibility

The mass spectrometry proteomics data have been deposited to the ProteomeXchange Consortium [1] via the PRIDE partner repository with the dataset identifier PXD001835″. [1] Vizcaíno JA, Deutsch EW, Wang R, Csordas A, Reisinger F, Ríos D, Dianes JA, Sun Z, Farrah T, Bandeira N, Binz PA, Xenarios I, Eisenacher M, Mayer G, Gatto L, Campos A, Chalkley RJ, Kraus HJ, Albar JP, Martinez-Bartolomé S, Apweiler R, Omenn GS, Martens L, Jones AR, Hermjakob H (2014). ProteomeXchange provides globally co-ordinated proteomics data submission and dissemination. Nature Biotechnol. 30(3):223-226. PubMed PMID:24727771.
