# Supplementary material for: Three-dimensional topology of the SMC2/SMC4 subcomplex from chicken condensin I revealed by cross-linking and molecular modelling
Source: Open Biol. 2015 Feb 25;5(2):150005. doi: 10.1098/rsob.150005 (PMC4345284; doi:10.1098/rsob.150005)
Supplement: SUPPLEMENTARY MATERIALS LEGENDS.docx [file rsob150005supp1.doc]

**SUPPLEMENTARY MATERIALS**

**Supplementary Figure Legends**

**Supplementary Figure 1.** **Further analysis of condensin and the SMC2-SMC4 sub-complex. (A)** Difference map showing only those linkage sites from band iii that were not seen in the putative cross-linked dimer of band i. **(B)** SDS-PAGE of titration of BS3 cross-linker ratio on SMC2/SMC4 complexes isolated from asynchronous and mitotic cells. The individual subunits resolved into bands as seen in lanes 1 and 6 (no cross-linker) disappear with increasing concentrations of cross-linker. A linkage product (asterisk) corresponding to the pentameric condensin complex (confirmed by mass spectrometry) is faintly visible and only for the mitotic but not the asynchronous sample. **(C)** Detailed map of cross-links in the SMC2/SMC4 subcomplex folded into an approximation of its 3D organisation, visualized using xiNET (www.crosslinkviewer.org). Head domains (brown), coiled-coils of SMC4 (red), coiled-coils of SMC2 (purple) are indicated. Cross-links between SMC2 and SMC4 (blue), and within SMC2 and SMC4 (green) are shown as dotted lines.

**Supplementary Figure 2.** **Mass spectrometry-coupled cross-linking analysis of cohesin complex. (A)** SDS-PAGE analysis of cohesin complex and BS3 cross-linked cohesin complex. Three products of cross-linking were observed (indicated by i, ii, iii), and the upper band was analyzed by mass spectrometry. Image shows an immunoblot of the cohesin complex using antibody recognising the Myc-tag. **(B)** NATIVE-PAGE analysis of condensin complexes isolated from DT40 cells. **(C)** Linkage map of cohesin complex representing interactions between the subunits, visualized using xiNET (www.crosslinkviewer.org). Green linkages are intramolecular and blue linkages are intermolecular. **(D)** Detailed map of coiled-coil arrangement of SMC1 and SMC3. Globular domain (brown), coiled-coils regions of SMC3 (red), coiled-coils regions of SMC1 (purple), linkage sites between SMC1 and SMC3 (blue), and within SMC1 and SMC3 (green) are indicated. Visualized using xiNET (www.crosslinkviewer.org).

**Supplementary Figure 3.** **Analysis of spectra for condensin and cohesin cross-links detected *in situ* in mitotic chromosomes. (A)** MS/MS spectra of cross-linked peptides connecting the centres of the coiled-coils of SMC2 (ESLKNAENELSSEK) and SMC4 (FTQLDLQDVKVR). **(B)** MS/MS spectra of cross-linked peptides linking the same site on the SMC4 coiled-coil (FTQLDLQDVKVR) to the other (paired) strand of the SMC2 coiled-coil (KQNLNSEENR). **(C)** MS spectrum indicating the CAP-H-CAP-H interaction. Both fragments contain the same N-terminal peptide of CAP-H, and must therefore come from different molecules. **(D)** MS/MS spectrum indicating interaction between the head of SMC1 (LIEIENF**K**SYK) and SA-2 (IIG**K**R). All spectra were plotted using xiSPEC (www.spectrumviewer.org).

**Supplementary Figure 4. Analysis of spectra for Histone-condensin cross-links detected *in vitro* in pull-downs. (A)** MS/MS spectrum of a cross-link indicating an interaction between the hinge domain of SMC4 (GEIIEQSGTMTGGGGK**V**MK) and N-terminal tail of H2A (S**S**RAGLQFPVGR). **(B)** A second cross-link between the hinge domain of SMC2 (TSQLEA**T**EK) and N-terminal tail of H2A.Z (AGGBAGK). All spectra plotted using xiSPEC (www.spectrumviewer.org).

**Supplementary Figure 5. Analysis of spectra for Histone-condensin cross-links detected *in situ* in mitotic chromosomes. (A)** MS/MS spectrum indicating interaction between the hinge domain of SMC4 (SG**S**ISGIHGR) and C-terminal tail of H2A (VTIAQGGVLPNIQAVLLP**K**K), and **(B)** between the head domain of SMC2 (AS**S**LQDLVYK) and C-terminal tail of H2A (VTIAQGGVLPNIQAVLLP**K**K). **(C)** MS/MS spectrum from *in situ* study indicating interaction between CAP-D2 (R**T**ALR) and the N-terminal tail of H4 (DNIQGIT**K**PAIR), and **(D)** interaction between the CAP-D2 (R**T**ALR) and the C-terminal tail of H4 (TV**T**AMDVVYALK). All spectra plotted using xiSPEC (www.spectrumviewer.org).

**Supplementary Figure 6.** **Condensin-Histone links visualized on a nucleosome core particle.** Cross-links detected *in vitro* between the chicken condensin subunits and histones, are shown mapped onto the crystal structure of a single nucleosome from *Xenopus laevis* (PDB:1AO) (94). See Figure 4 for a diagram of the cross-links observed *in situ*.

**Supplementary Table 1.** **List of all cross-linked condensin and cohesin peptides identified in this study.**

**Supplementary Table 2.** **Inclusion list used in the attempt to identify cross-links in condensin cross-linked in intact mitotic chromosomes.** The list contains m/z values of all cross-links detected in our study of purified condensin and cohesin. File format is an Excel spread sheet.

**Supplementary Data File 1 (.tgz)**

Low-resolution model of a full-length rod-like chicken SMC2-SMC4 core complex. Upon uncompressing, the resulting folder "chickenSMC2SMC4core.Barysz_et_al_2015" includes the atomic 3D coordinates (PDB format) (file name: Barysz_et_al_2015.chickenSMC2SMC4_draft_struc.pdb), the same model pre-rendered for easy visualization for UCSF Chimera (file name: Barysz_et_al_2015.chickenSMC2SMC4_draft_struc.py), and pre-rendered for PyMOL (file name: Barysz_et_al_2015.chickenSMC2SMC4_draft_struc.pse), and instructions for downloading the programs, accessing and opening the pre-rendered files to view the structure interactively.
