# Supplementary material for: Three-dimensional topology of the SMC2/SMC4 subcomplex from chicken condensin I revealed by cross-linking and molecular modelling
Source: Open Biol. 2015 Feb 25;5(2):150005. doi: 10.1098/rsob.150005 (PMC4345284; doi:10.1098/rsob.150005)
Supplement: Barysz et al. Supplementary PDFs [file rsob150005supp2.pdf]

A Map of cross-links specific for band iii (cross-links also seen in band i not shown)

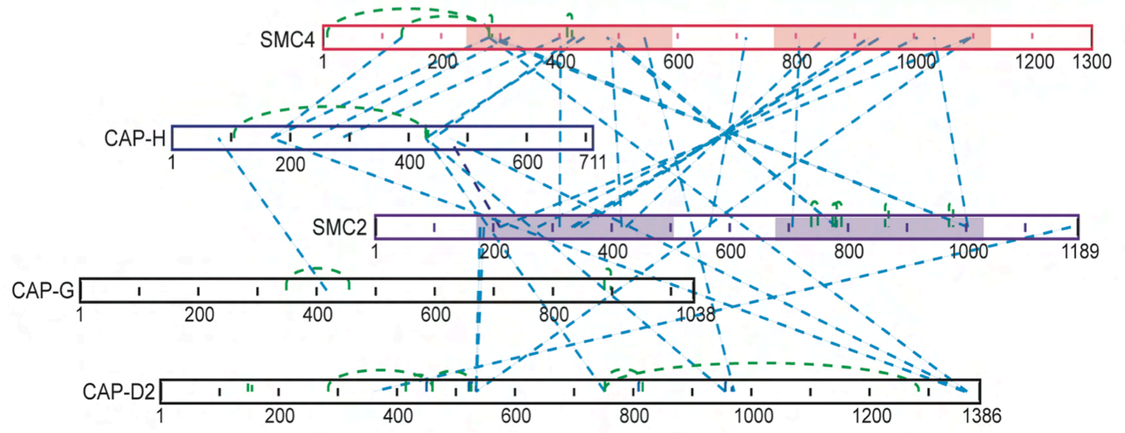

B

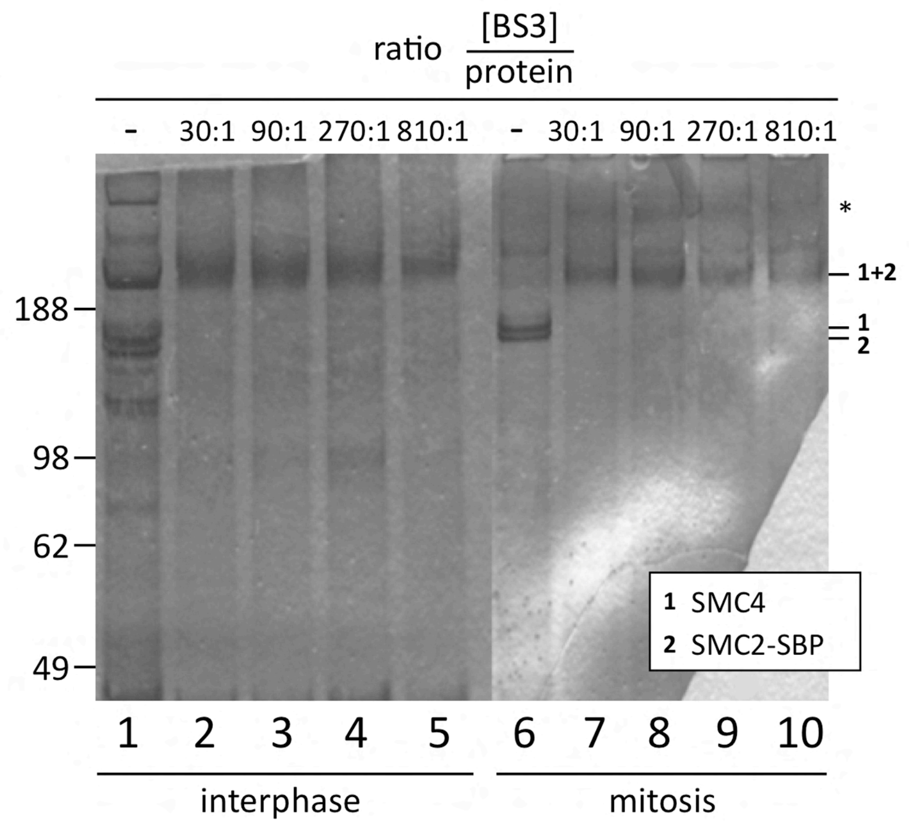

C SMC2/SMC4 cross-links displayed on a topological representation of the complex

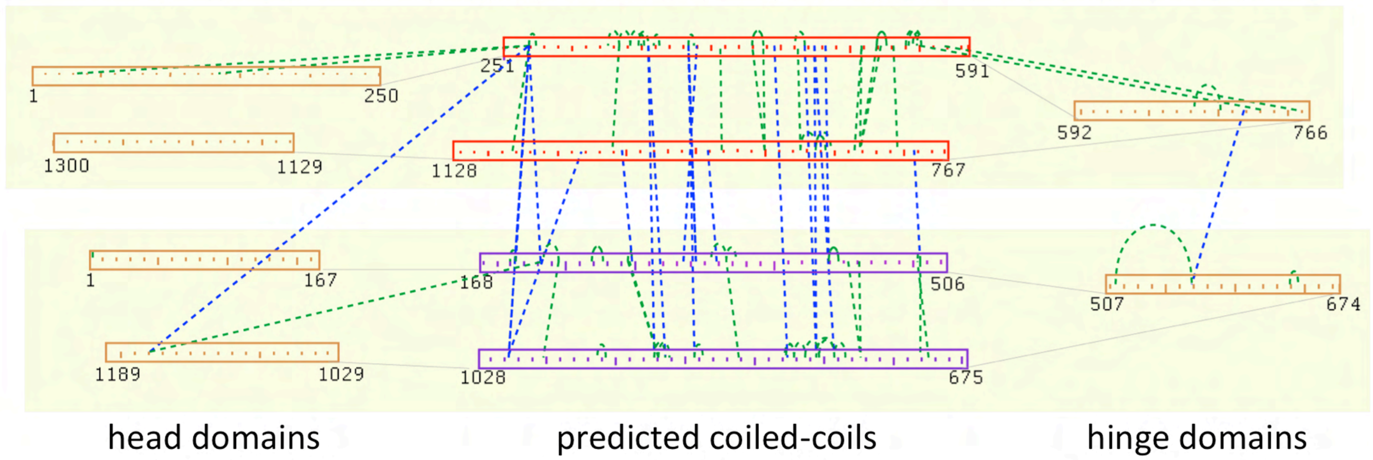

Barysz et al. Supplementary Figure 1

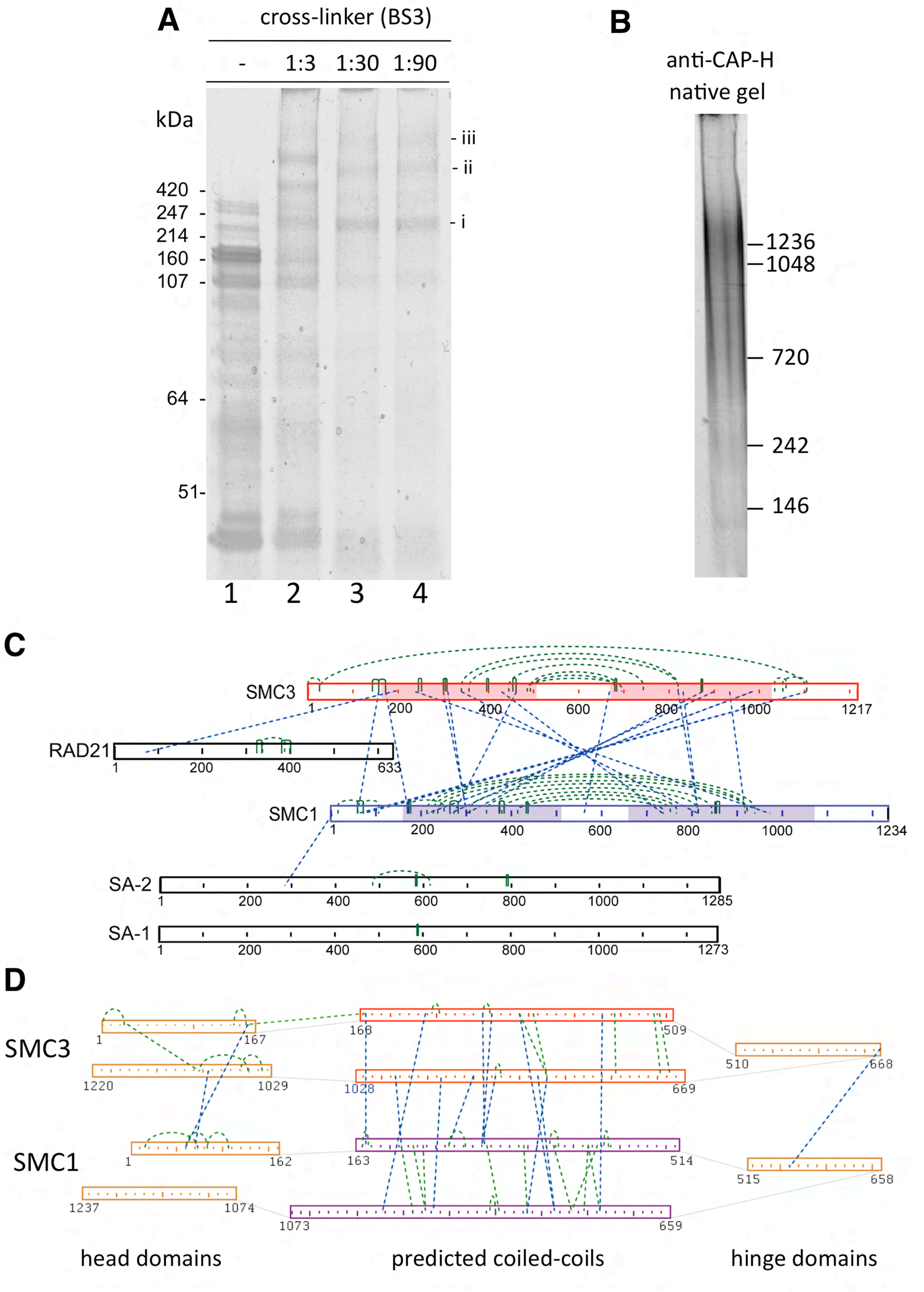

Barysz et al. Supplementary Figure 2

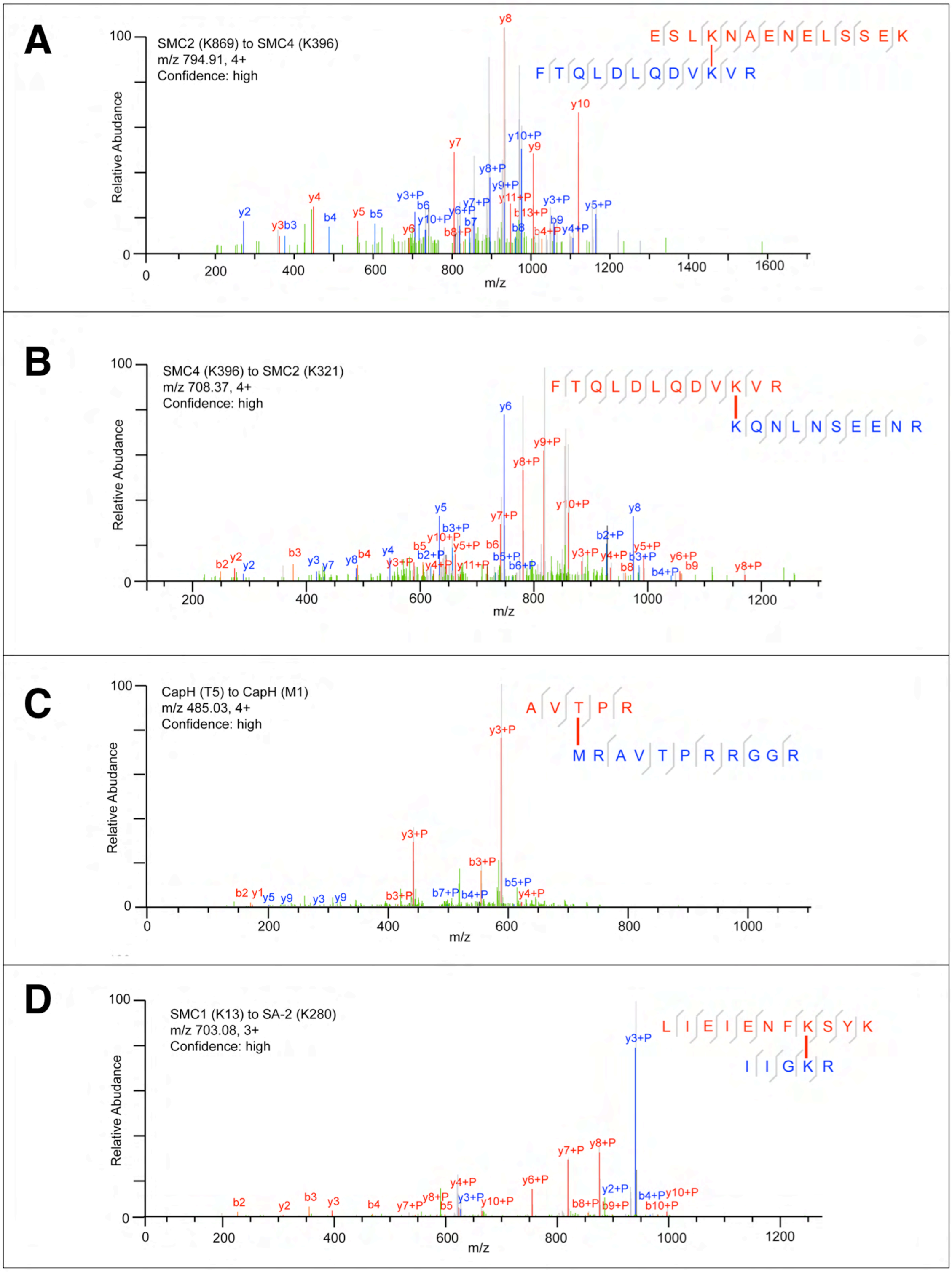

Barysz et al. Supplementary Figure 3

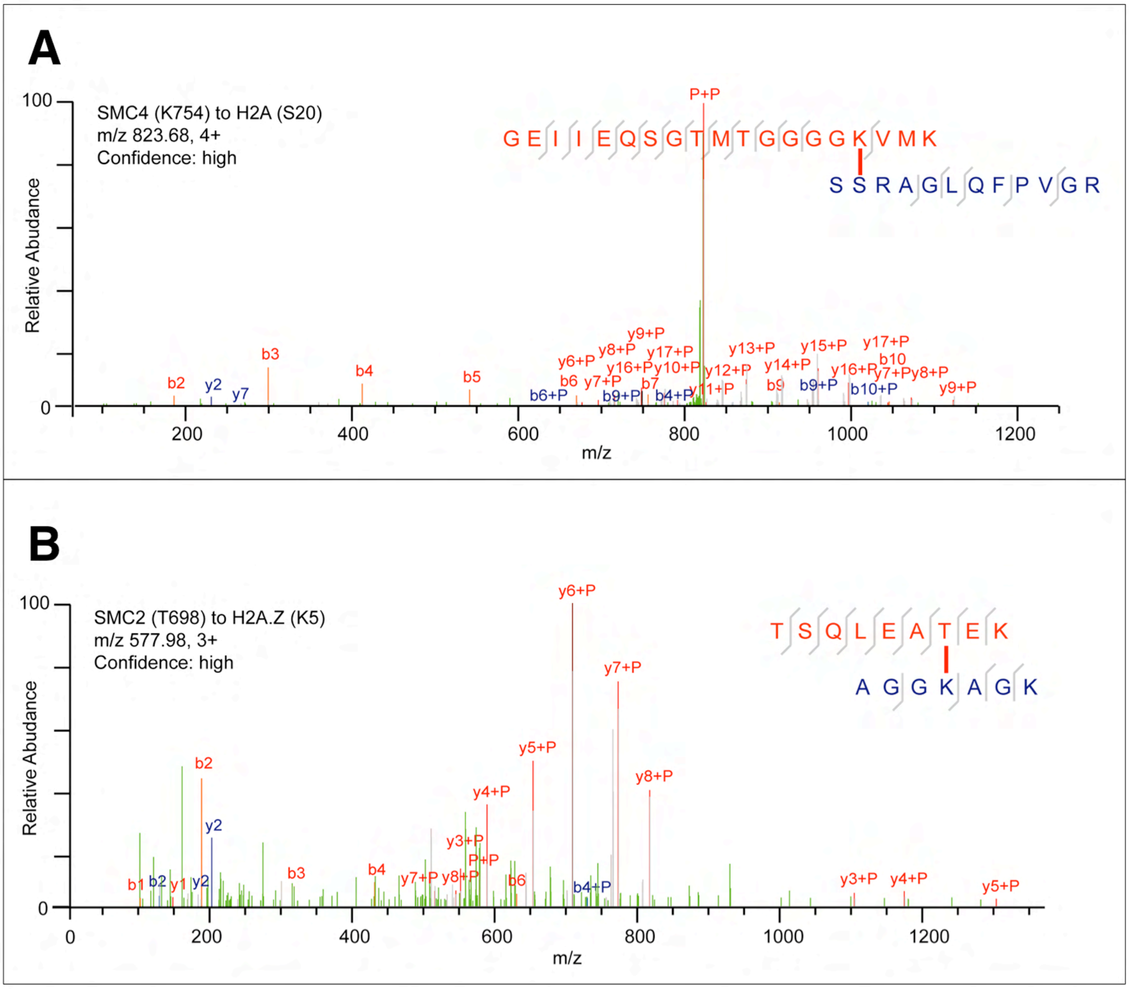

Barysz et al. Supplementary Figure 4

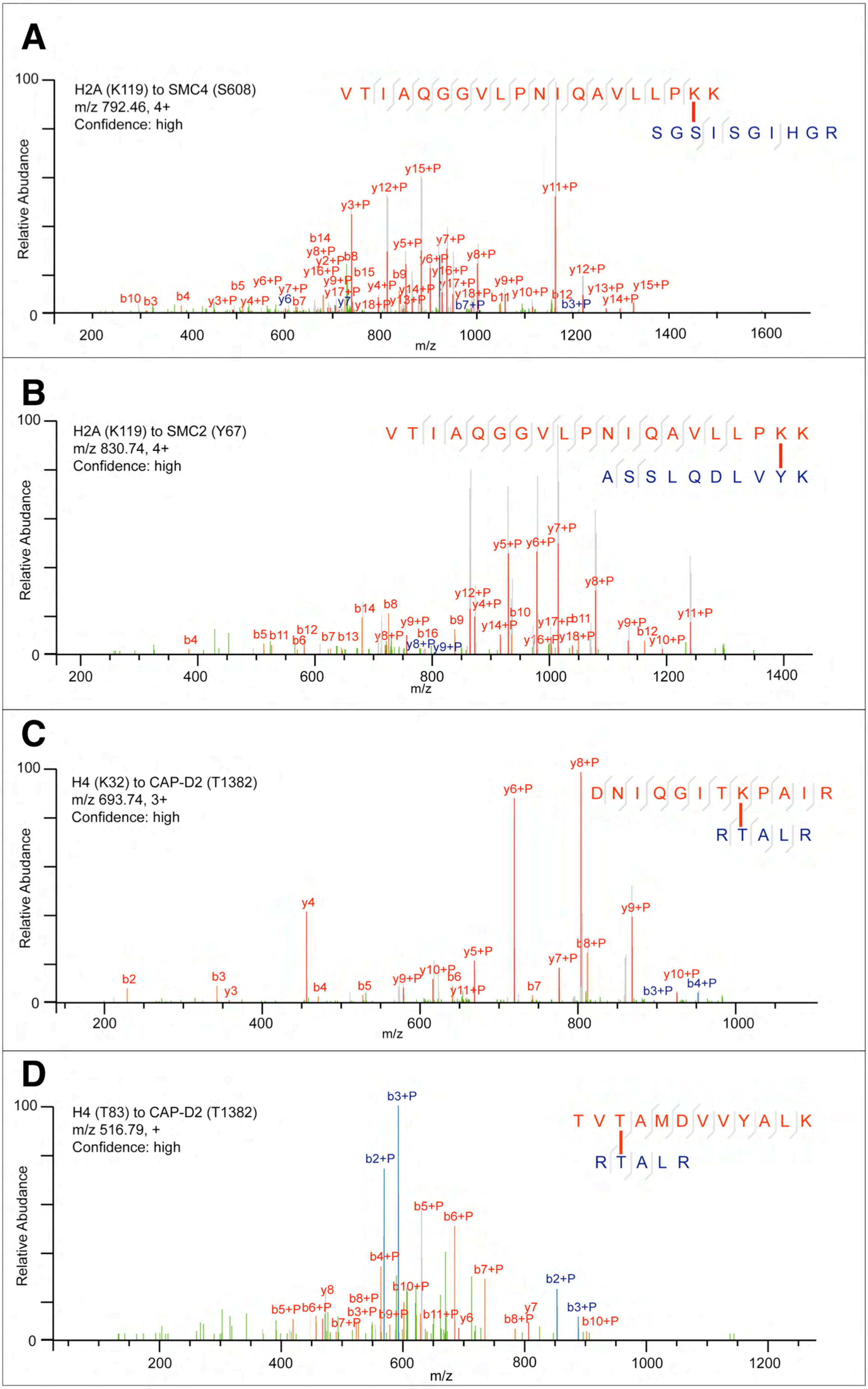

Barysz et al. Supplementary Figure 5

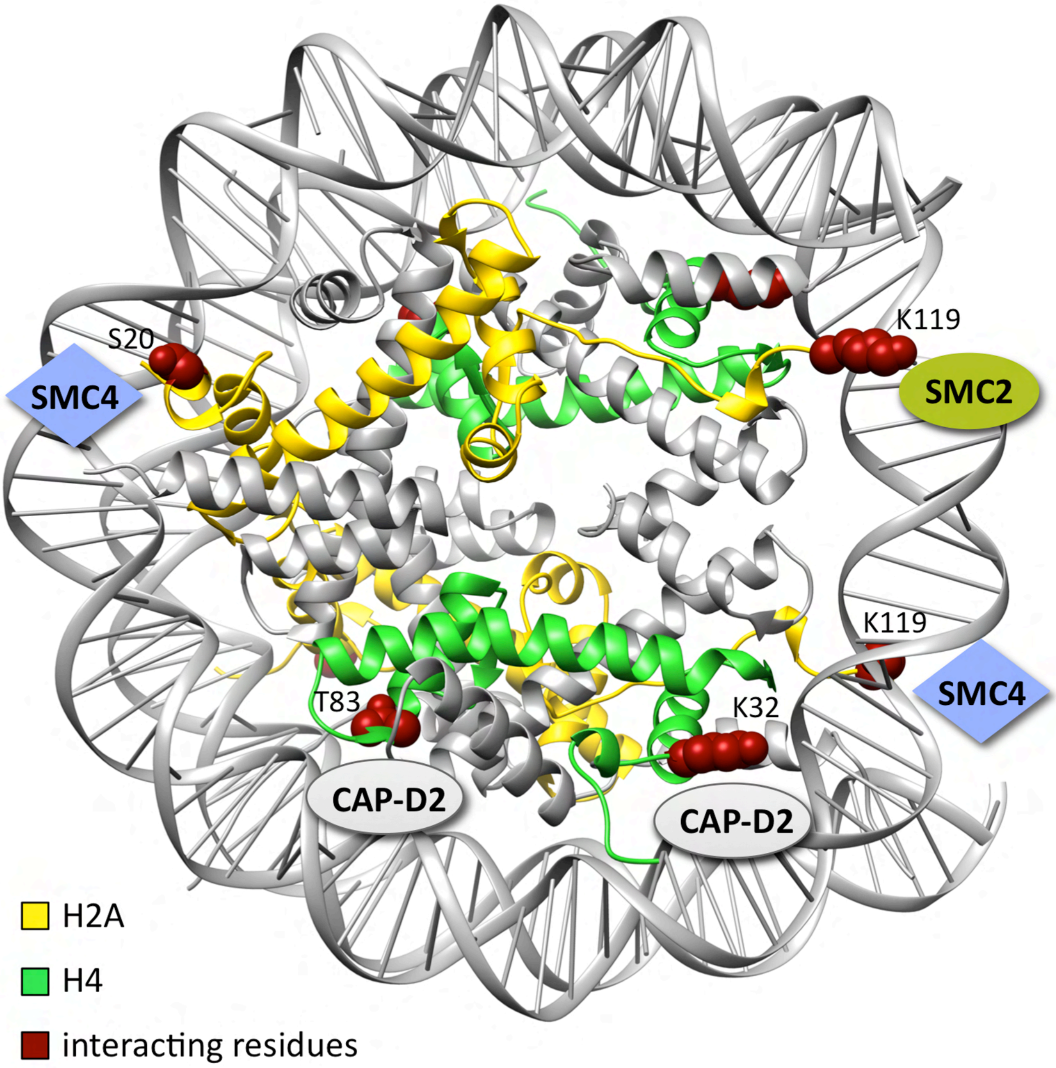

Barysz et al. Supplementary Figure 6

Supplementary Table I. Summary of cross-links observed in this study

BAND i

Protein1	Peptide Position 1	Peptide 1	Pep 1 Link pos	Protein2	Peptide Position 2	Peptide 2	Pep 2 Link pos
SMC4	535	AAIKDIDIKLPTAEQELK	4	SMC4	521	EALMoxTTSETLKQR	11
CAP-D2	1279	AASKPETK	4	CAP-D2	1358	NNKR	3
SMC2	411	AATEAKQAQMK	6	SMC2	422	LKYAQQELK	2
SMC2	411	AATEAKQAQMoxK	6	SMC2	422	LKYAQQELK	2
SMC4	448	AKDKEEEK	4	SMC4	434	NISDATSKK	8
SMC2	618	AMoxEYVFGTTLVCcmSSMoxDNAKK	19	SMC2	638	VTFDKR	5
CAP-D2	1362	AQKSHR	3	CAP-D2	1279	AASKPETK	4
CAP-D2	1362	AQKSHR	3	CAP-D2	1358	NNKR	3
SMC4	919	AQVAIKTAHR	6	SMC4	929	NLKK	3
SMC2	214	ASYLEYQKMoxTR	8	CAP-H	198	KHSFK	1
SMC2	214	ASYLEYQKMoxTR	8	SMC2	209	LKEAR	2
SMC4	786	AVQCcmEEEKFQLEEDITK	16	SMC4	803	LQKNVR	3
SMC4	417	DKEKVEELKNVPSSSEK	9	SMC4	929	NLKK	3
CAP-G	943	DVPHTPVTEVKSK	11	CAP-G	960	LKSTR	2
SMC4	521	EALMoxTTSETLKQR	11	SMC4	535	AAIKDIDIK	4
CAP-D2	952	EEEKTK	4	CAP-D2	970	KRPR	1
SMC2	275	ELAQQIEETEK	9	SMC2	286	KNNEEFGAK	1
SMC4	854	ELEKVLNSYK	4	SMC4	864	KDYER	1
CAP-G	524	EQITELEHTK	4	SMC4	409	KLEK	1
CAP-G	524	EQITELEHTKTNLIK	10	CAP-G	493	LAEIKVK	5
CAP-G	524	EQITELEHTKTNLIK	10	CAP-G	493	LAEIKVK	5

SMC2	865	ESLKNAENELSSEK	4	SMC4	386	FTQLDLQDVKVR	10
SMC2	507	FEYKNPEK	4	SMC2	562	ILEKGQLK	4
SMC2	507	FEYKNPEK	4	SMC2	561	KILEKGQLK	5
SMC4	386	FTQLDLQDVKVR	10	SMC2	865	ESLKNAENELSSEK	4
SMC4	386	FTQLDLQDVKVR	10	SMC2	320	KQNLNSEENR	1
SMC4	386	FTQLDLQDVKVR	10	SMC2	320	KQNLNSEENRLK	1
CAP-H	431	HKSSADSEK	2	CAP-H	427	FKPR	2
CAP-H	431	HKSSADSEKENK	2	CAP-H	427	FKPR	2
CAP-H	603	IAIQYAKTAK	7	CAP-H	613	KMoxDMoxK	1
CAP-H	603	IAIQYAKTAK	7	CAP-H	613	KMoxDMoxKR	1
SMC4	372	IDKITVLIEENKNK	3	SMC4	364	ALKDLEKK	7
SMC4	372	IDKITVLIEENKNK	3	SMC4	367	DLEKK	4
CAP-G	452	IIKDDAR	3	CAP-G	729	LISAKLLSR	5
SMC2	354	IKEGLNGLQEESK	2	SMC2	348	EKEIKK	2
SMC2	562	ILEKGQLK	4	SMC2	507	FEYKNPEK	4
CAP-D2	949	ILREEEKTK	7	CAP-D2	958	KHKER	3
SMC2	336	IMoxQEEFKAFTSK	7	SMC2	330	LKELIK	2
SMC4	330	INDLETQKENIQKETKDINEK	16	SMC4	1006	EALNIKFK	6
SMC4	330	INDLETQKENIQKETKDINEK	16	SMC4	351	SSKLADEMoxK	3
SMC2	178	ITAHKTIEK	5	SMC2	172	MoxYECcmKK	5
CAP-G	973	KAAVGTR	1	CAP-G	960	LKSTR	2
CAP-G	973	KAAVGTR	1	CAP-G	980	SVCcmR	1
SMC2	767	KAEEEYK	1	SMC2	760	KTEESQR	1
SMC4	563	KEELNAQDLVR	1	SMC4	555	ENKLEKLK	6
SMC4	563	KEELNAQDLVR	1	SMC4	558	LEKLK	3
SMC2	261	KIFESMoxAENEK	1	SMC2	248	STNALKEAQANKK	6
SMC2	261	KIFESMoxAENEKK	1	SMC2	248	STNALKEAQANKK	6
SMC2	353	KIKEGLNGLQEESKK	3	SMC2	348	EKEIK	2

SMC2	561	KILEKGQLK	5	SMC2	507	FEYKNPEK	4
CAP-H	197	KKHSFK	2	CAP-H	193	VQTK	3
SMC2	260	KKIFESMoxAENEK	1	SMC2	248	STNALKEAQANK	6
CAP-G	227	KLAYEVLAEK	1	CAP-G	210	TLPKIVGR	4
CAP-G	227	KLAYEVLAEK	1	CAP-G	218	TMoxDVKEAVR	5
SMC2	482	KQLSSEISSLR	1	SMC4	803	LQKNVR	3
SMC2	482	KQLSSEISSLR	1	SMC4	803	LQKNVR	3
SMC2	482	KQLSSEISSLR	1	SMC4	803	LQKNVR	3
SMC2	320	KQNLNSEENR	1	SMC4	386	FTQLDLQDVKVR	10
SMC2	1176	KQPLSEASNNKDE	1	SMC2	1170	HCcmQLKK	5
CAP-G	488	KQQIKLAEIK	5	CAP-G	498	VKLFEAK	2
SMC4	354	LADEMKSK	6	SMC4	364	ALKDLEK	3
CAP-D2	799	LAEEVCcmNAISK	10	CAP-D2	810	LASNSKPAEGK	6
CAP-G	493	LAEIKVK	5	CAP-G	452	IIKDDARR	3
CAP-D2	810	LASNSKPAEGK	6	CAP-D2	821	NSVPFR	2
SMC4	689	LIDLVKVEDK	6	SMC4	676	NLQKIPTPENAPR	4
CAP-G	729	LISAKLLSR	5	CAP-G	452	IIKDDAR	3
CAP-G	152	LKDKFSNVR	2	CAP-G	218	TMoxDVKEAVR	5
SMC4	456	LKQVMoxSSLQEETR	2	SMC2	789	EIKNAQQK	3
SMC4	456	LKQVMoxSSLQEETR	2	SMC2	787	GKEIKNAQQK	5
SMC4	456	LKQVMoxSSLQEETR	2	SMC2	802	KKADDSSR	1
SMC2	422	LKYAQQELK	2	SMC2	411	AATEAKQAQMK	6
SMC2	467	LKYEEAEQEAHLAK	2	SMC4	803	LQKNVR	3
SMC2	467	LKYEEAEQEAHLAK	2	SMC2	706	NMoxAEKYQHLK	5
SMC2	467	LKYEEAEQEAHLAK	2	SMC2	711	YQHLKQQWEMoxK	5
CAP-D2	1062	LLFTMoxMoxEK	4	CAP-D2	1070	SPLPVVR	1
CAP-D2	530	LTQKALCcmR	4	CAP-D2	455	LQEMoxKDR	5
CAP-D2	530	LTQKALCcmR	4	CAP-D2	455	LQEMoxKDR	5
SMC2	439	MoxDGSYKEDQEAFEAIRK	6	SMC2	433	QAEVKK	5

SMC4	759	MoxGSSVVTDVSPEEVNR	10	SMC4	593	GKVLEALLEQKR	2
SMC2	792	NAQQKLNSAK	5	SMC2	787	GKEIK	2
SMC2	792	NAQQKLNSAK	5	SMC2	803	KADDSSR	1
SMC2	112	NKYLINGMoxNASNNR	2	SMC2	68	NGQAGVNKATVSITFDNSDKK	8
SMC4	676	NLQKIPTPENAPR	4	SMC4	689	LIDLVKVEDK	6
CAP-D2	821	NSVPFR	2	CAP-D2	810	LASNSKPAEGK	6
SMC4	577	QKVEEAK	2	SMC2	566	GQLKHR	4
SMC2	740	QQEDLLALKK	9	SMC2	424	YAQQELKTK	7
CAP-G	489	QQIKLAEIK	4	CAP-G	498	VKLFEAK	2
CAP-H	644	QSDTSVVTGEKAFSSITK	11	SMC4	1	ASRSTK	1
CAP-D2	932	QVAHLEVSVSAELR	8	CAP-H	655	AFSSITKELLHR	7
SMC4	269	RVETLNEQRGEKLNR	12	SMC4	1095	KEELYLKR	7
SMC4	269	RVETLNEQRGEKLNR	12	SMC4	1094	RKEELYLK	2
CAP-D2	963	SDSSTIK	5	CAP-D2	970	KRPR	1
CAP-D2	963	SDSSTIKK	7	CAP-D2	959	HKER	2
CAP-D2	963	SDSSTIKK	7	CAP-D2	949	ILREEEKTK	7
CAP-H	374	SFTTVCcmESKK	9	CAP-H	427	FKPR	2
CAP-H	476	SILENQNVK	1	CAP-H	469	AALTLSK	6
SMC4	362	SKALKDLEK	2	SMC4	354	LADEMoxKSK	6
SMC4	493	SKMoxDVAQAELDIYLTR	2	SMC2	411	AATEAKQAQMoxK	6
CAP-H	433	SSADSEK	1	CAP-H	427	FKPR	2
CAP-H	433	SSADSEKENK	7	CAP-H	427	FKPR	2
CAP-H	433	SSADSEKENKK	7	CAP-H	427	FKPR	2
CAP-H	633	STEAEIEDVEKQSDTSVVTGEK	11	CAP-H	655	AFSSITKELLHR	7
SMC4	200	STFKDVGILLR	4	SMC4	130	AQKIR	3
SMC2	248	STNALKEAQANK	6	SMC2	241	AEEIKDR	5

SMC2	248	STNALKEAQANK	6	SMC2	241	AEEIKDR	5
CAP-G	980	SVCcmR	1	CAP-G	973	KAAVGTR	1
CAP-H	203	TIEQNLNNINVTEANRR	1	CAP-H	199	HSFK	2
CAP-H	467	TKAALTLSK	2	CAP-G	154	DKFSNVR	2
SMC2	885	TKDIKAK	5	SMC2	895	IEKYR	3
SMC2	1158	TKFVDGISTVSR	2	SMC2	209	LKEAR	2
CAP-G	210	TLPKIVGR	4	CAP-G	452	IIKDDAR	3
CAP-G	210	TLPKIVGR	4	CAP-G	218	TMoxDVKEAVR	5
CAP-G	218	TMoxDVKEAVR	5	CAP-H	427	FKPR	2
SMC4	897	TQQDKVDKINQEIDECcmTSAITK	5	SMC4	434	NISDATSKK	8
SMC2	691	TSQLEATEKELANLK	9	SMC2	467	LKYEEAEQEAHLAKK	14
SMC2	691	TSQLEATEKELANLK	9	SMC2	467	LKYEEAEQEAHLAKKK	14
SMC4	7	TSTAAHQKGR	8	SMC4	278	GEKLNR	3
H4	80	TVTAMoxDVVYALKR	12	H2A	36	KGNYAER	1
CAP-H	151	VDAVHADTYK	8	CAP-H	161	VLGGLGKESAPTK	7
CAP-H	151	VDAVHADTYKVLGGLGK	10	CAP-H	427	FKPR	2
CAP-H	151	VDAVHADTYKVLGGLGK	10	CAP-H	193	VQTKK	4
SMC4	695	VEDKSFSPAFYFALR	4	SMC4	675	KNLQK	1
SMC4	695	VEDKSFSPAFYFALR	4	SMC4	676	NLQKIPTPENAPR	4
SMC4	421	VEELKNVPSSSEK	5	SMC4	929	NLKK	3
CAP-G	550	VEKNDPETLLK	3	CAP-G	452	IIKDDARR	3
SMC4	270	VETLNEQRGEKLNR	11	SMC4	1094	RKEELYLKR	8
SMC2	1071	VGLGDIWKENLTELSGGQR	8	SMC2	209	LKEAR	2
SMC2	273	VKELAQQIEETEK	11	SMC2	286	KNNEEFGAK	1
CAP-G	248	VKLLQQGLNDR	2	CAP-G	452	IIKDDAR	3

CAP-G	248	VKLLQQGLNDR	2	CAP-G	218	TMoxDVKEAVR	5
CAP-H	161	VLGGLGKESAPTK	7	CAP-H	193	VQTKK	4
SMC4	869	VSEQAGKMESEVKR	7	SMC4	864	KDYER	1
SMC4	869	VSEQAGKMoxESEVKR	7	SMC4	473	EKEGKEK	5
SMC4	869	VSEQAGKMoxESEVKR	7	SMC4	864	KDYER	1
SMC4	733	VVTLKGEIIEQSGTMoxT GGGGK	5	SMC4	754	VMoxKGR	3
SMC2	424	YAQQELKTK	7	SMC2	433	QAEVKK	5
SMC4	509	YNTAVSQLDEAKEALMo xTTSETLK	12	SMC4	831	LKGQVK	2
SMC4	509	YNTAVSQLDEAKEALMo xTTSETLKQR	23	SMC4	831	LKGQVK	2
SMC2	711	YQHLKQQWEMoxK	5	SMC2	467	LKYEEAEQEAHLAK	2

BAND ii

Protein1	Peptide Position 1	Peptide 1	Pep 1 Link pos	Protein2	Peptide Position 2	Peptide 2	Pep 2 Link pos
SMC2	411	AATEAKQAQMK	6	SMC2	422	LKYAQQELK	2
SMC2	411	AATEAKQAQMoxK	6	SMC2	422	LKYAQQELKbs3ohTK	2
SMC4	364	ALKDLEK	3	SMC4	354	LADEMoxKSK	6
SMC2	214	ASYLEYQKMoxTR	8	SMC2	209	LKEAR	2
SMC4	521	EALMoxTTSETLKQR	11	SMC4	535	AAIKDIDIK	4
SMC2	275	ELAQQIEETEK	9	SMC2	286	KNNEEFGAK	1
SMC4	854	ELEKVLNSYK	4	SMC4	864	KDYER	1
SMC2	865	ESLKNAENELSSEK	4	SMC4	386	FTQLDLQDVKVR	10
SMC2	865	ESLKNAENELSSEK	4	SMC4	386	FTQLDLQDVKVR	10

SMC2	507	FEYKNPEK	4	SMC2	562	ILEKGQLK	4
SMC4	386	FTQLDLQDVKVR	10	SMC2	865	ESLKNAENELSSEK	4
SMC4	278	GEKLNR	3	SMC4	1095	KEELYLKR	7
SMC2	262	IFESMoxAENEKK	10	SMC2	892	SAKIEK	3
SMC2	562	ILEKGQLK	4	SMC2	507	FEYKNPEK	4
CAP-G	227	KLAYEVLAEK	1	CAP-G	218	TMoxDVKEAVR	5
SMC2	760	KTEESQR	1	SMC2	767	KAEEEYK	1
SMC4	354	LADEMKSK	6	SMC4	364	ALKDLEK	3
SMC4	456	LKQVMoxSSLQEETR	2	SMC2	802	KKADDSSR	2
SMC2	422	LKYAQQELKbs3nh2TK	2	SMC2	411	AATEAKQAQMoxK	6
CAP-D2	439	LSCcmSDLAEPLKK	11	CAP-D2	455	LQEMoxKDR	5
CAP-D2	530	LTQKALCcmR	4	CAP-D2	521	KLNYK	1
CAP-D2	530	LTQKALCcmR	4	CAP-D2	455	LQEMoxKDR	5
SMC2	779	MoxKNAEAER	2	SMC2	767	KAEEEYKALENK	7
SMC2	792	NAQQKLNSAK	5	SMC2	787	GKEIK	2
SMC2	792	NAQQKLNSAK	5	SMC2	803	KADDSSR	1
SMC2	792	NAQQKLNSAK	5	SMC2	802	KKADDSSR	2
SMC2	406	NEISKAATEAK	5	SMC2	760	KTEESQR	1
SMC4	434	NISDATSKK	8	SMC4	448	AKDKEEEK	4
CAP-H	433	SSADSEKENK	1	CAP-H	427	FKPR	2
SMC2	248	STNALKbs3ohEAQANK	2	SMC2	260	KKIFESMoxAENEK	2
SMC2	248	STNALKEAQANK	6	SMC2	241	AEEIKDR	5
SMC2	248	STNALKEAQANKbs3nh2<br>K	6	SMC2	241	AEEIKDR	5
SMC2	248	STNALKEAQANKbs3ohK	6	SMC2	241	AEEIKDR	5
SMC2	248	STNALKEAQANKK	6	SMC2	241	AEEIKDR	5
CAP-G	210	TLPKIVGR	4	CAP-G	218	TMoxDVKEAVR	5
CAP-G	218	TMDVKEAVR	5	CAP-H	427	FKPR	2
CAP-G	218	TMoxDVKEAVR	5	CAP-H	427	FKPR	2
CAP-G	218	TMoxDVKEAVR	5	CAP-H	427	FKPR	2

SMC2	691	TSQLEATEKELANLK	9	SMC2	682	DAEDELKIK	7
SMC2	308	VDAKVR	4	SMC2	314	SDLDHR	1
CAP-H	161	VLGGLGKESAPTK	7	CAP-H	193	VQTKK	4
SMC4	869	VSEQAGKMoxESEVKR	7	SMC4	864	KDYER	1
SMC2	424	YAQQELKTK	7	SMC2	433	QAEVKK	5

BAND iii

Protein1	Peptide Position 1	Peptide 1	Pep 1 Link pos	Protein2	Peptide Position 2	Peptide 2	Pep 2 Link pos
CAP-D2	1279	AASKPETK	4	CAP-D2	1208	QTESLVEKLCcmQR	8
SMC2	411	AATEAKQAQMK	6	SMC4	480	ELMEFCcmKTVNDAR	7
SMC2	411	AATEAKQAQMK	6	SMC2	422	LKYAQQELK	2
SMC2	411	AATEAKQAQMoxK	6	SMC2	422	LKYAQQELK	2
SMC2	241	AEEIKDR	5	SMC2	933	LLKEYK	3
SMC2	241	AEEIKDR	5	SMC2	248	STNALKEAQANKK	6
SMC2	774	ALENKMK	5	SMC2	787	GKEIK	2
SMC2	774	ALENKMK	5	SMC2	767	KAEEEYK	1
SMC4	364	ALKDLEK	3	SMC4	354	LADEMoxKSK	6
SMC4	130	AQKIR	3	SMC4	278	GEKLNR	3
SMC4	919	AQVAIKTAHR	6	SMC2	343	AFTSKEK	5
CAPG	342	CcmLCcmEHLKSK	7	CAP-G	452	IIKDDAR	3
SMC2	682	DAEDELKIK	7	SMC2	507	FEYKNPEK	4
SMC4	539	DIDIKLPTAEQELK	5	SMC4	794	FQLEEDITKLQK	9
SMC4	346	DINEKSSK	5	SMC4	330	INDLETQKENIQKETK	8
SMC4	450	DKEEEKLK	6	SMC4	443	ELLEKAK	5
SMC4	417	DKEKVEELK	4	SMC4	932	KSEDSVLR	1
SMC4	710	DTLVVKDLEDATR	6	SMC2	562	ILEKGQLK	4

SMC4	521	EALMoxTTSETLKQR	11	SMC4	535	AAIKDIDIK	4
SMC4	521	EALMTTSETLKQR	11	SMC4	535	AAIKDIDIK	4
SMC4	475	EGKEKELMEFCcmK	5	SMC4	469	EIQKEK	4
CAP-D2	279	EIGQKCcmPQELAR	5	CAP-D2	455	LQEMKDR	5
SMC4	950	EIKDLTEELTTLEDK	3	SMC4	940	TEKEIGDNEK	3
SMC4	478	EKELMEFCcmK	2	SMC4	469	EIQKEK	4
SMC4	478	EKELMEFCcmK	2	SMC2	779	MKNAEAER	2
SMC4	478	EKELMEFCcmK	2	SMC2	779	MoxKNAEAER	2
SMC4	478	EKELMoxEFCcmK	2	SMC4	469	EIQKEK	4
SMC4	478	EKELMoxEFCcmKTVNDAR	9	SMC2	779	MoxKNAEAER	2
SMC4	398	EKLKHAK	4	CAP-H	285	SLLEK	1
SMC2	700	ELANLKNMAEK	6	SMC4	803	LQKNVR	3
SMC2	275	ELAQQIEETEK	9	SMC2	286	KNNEEFGAK	1
SMC4	837	ELEANVTAAAPDKNKQK	13	SMC2	422	LKYAQQELK	2
SMC2	332	ELIKIMQEEFK	4	SMC4	940	TEKEIGDNEK	3
SMC4	480	ELMEFCcmKTVNDAR	7	SMC2	411	AATEAKQAQMK	6
SMC4	480	ELMoxEFCcmKTVNDAR	7	SMC2	779	MKNAEAER	2
SMC2	851	EQVSALEAEAVKTR	12	SMC2	865	ESLKNAENELSSEK	4
SMC2	188	ESKLDEIR	3	SMC2	183	TIEKK	4
SMC2	865	ESLKNAENELSSEK	4	SMC4	386	FTQLDLQDVKVR	10
SMC4	343	ETKDINEK	3	SMC4	351	SSKLADEMK	3
SMC2	507	FEYKNPEK	4	SMC2	562	ILEKGQLK	4
CAP-D2	748	FTEKTQCcmSPLER	4	CAP-D2	1279	AASKPETK	4
CAP-D2	748	FTEKTQCcmSPLER	4	CAP-D2	810	LASNSKPAEGK	6
SMC4	386	FTQLDLQDVKVR	10	SMC2	865	ESLKNAENELSSEK	4
SMC4	386	FTQLDLQDVKVR	10	SMC2	320	KQNLNSEENR	1
SMC4	738	GEIIEQSGTMoxTGGGGKVMK	16	SMC4	759	MGSSVVTDVSPEEVNR	4

SMC4	738	GEIIEQSGTMTGGGGKV MK	16	H2A	18	SSRAGLQFPVGR	2
SMC4	278	GEKLNR	3	CAP-D2	1362	AQKSHR	3
SMC4	278	GEKLNR	3	SMC4	284	VKMVEK	2
SMC4	278	GEKLNR	3	SMC2	997	YNDLMKK	6
SMC2	787	GKEIKNAQQK	5	SMC2	797	LNSAKK	5
CAP-H	603	IAIQYAKTAK	7	CAP-H	613	KMDMK	1
CAP-H	603	IAIQYAKTAK	7	CAP-H	613	KMDMoxK	1
CAP-H	603	IAIQYAKTAK	7	CAP-H	613	KMoxDMK	1
CAP-H	603	IAIQYAKTAK	7	CAP-H	613	KMoxDMoxK	1
CAP-H	603	IAIQYAKTAK	7	CAP-H	614	MDMKR	4
SMC2	262	IFESMAENEKK	10	SMC2	892	SAKIEK	3
SMC2	354	IKEGLNGLQEESK	2	CAP-H	197	KKHSFK	2
SMC2	354	IKEGLNGLQEESKK	2	SMC2	348	EKEIKK	2
SMC2	689	IKTSQLEATEK	2	SMC2	467	LKYEEAEQEAHLAKK	14
SMC2	689	IKTSQLEATEK	2	SMC2	469	YEEAEQEAHLAKK	12
SMC2	562	ILEKGQLK	4	SMC2	507	FEYKNPEK	4
SMC4	330	INDLETQKENIQK	8	SMC4	343	ETKDINEK	3
SMC4	905	INQEIDECcmTSAITKAQ VAIK	14	SMC4	434	NISDATSKK	8
SMC2	178	ITAHKTIEK	5	SMC2	172	MYECcmKK	5
SMC4	534	KAAIK	1	SMC4	831	LKGQVK	2
SMC2	767	KAEEEYKALENK	7	SMC2	779	MKNAEAER	2
SMC4	1095	KEELYLKR	7	SMC4	278	GEKLNR	3
SMC2	261	KIFESMAENEK	1	SMC2	248	STNALKEAQANK	6
SMC2	261	KIFESMAENEKK	1	SMC2	248	STNALKEAQANKK	6
SMC2	261	KIFESMoxAENEK	1	SMC2	248	STNALKEAQANK	6
SMC2	177	KITAHK	1	SMC2	183	TIEKK	4
CAP-D2	521	KLNYK	1	CAP-D2	455	LQEMKDR	5
SMC2	438	KMDGSYKEDQEAFEAIR	1	SMC2	740	QQEDLLALKK	9

SMC2	482	KQLSSEISSLR	1	SMC2	700	ELANLKNMAEK	6
SMC2	482	KQLSSEISSLR	1	SMC4	803	LQKNVR	3
SMC2	320	KQNLNSEENR	1	SMC2	330	LKELIK	2
SMC2	1176	KQPLSEASNNKDE	1	CAP-D2	358	GTRDNFLK	2
SMC2	1176	KQPLSEASNNKDE	1	SMC2	1170	HCcmQLKK	5
SMC4	354	LADEMKSK	6	SMC4	362	SKALKDLEK	2
SMC4	354	LADEMoxKSK	6	SMC4	364	ALKDLEK	3
SMC4	354	LADEMoxKSK	6	SMC4	362	SKALK	2
CAP-D2	810	LASNSKPAEGK	6	CAP-D2	799	LAEEVCcmNAISK	10
SMC4	410	LEKQLQK	3	SMC4	417	DKEKVEELK	4
CAP-D2	411	LKDKSVVVVK	4	CAP-D2	455	LQEMKDR	5
SMC4	400	LKHAK	2	SMC2	308	VDAKVR	4
SMC4	561	LKKEELNAQDLVR	2	SMC4	555	ENKLEK	3
SMC4	456	LKQVMoxSSLQEETR	2	SMC2	789	EIKNAQQK	3
SMC4	456	LKQVMoxSSLQEETR	2	SMC2	787	GKEIKNAQQK	5
SMC4	456	LKQVMSSLQEETR	2	SMC2	789	EIKNAQQK	3
SMC2	467	LKYEEAEQEAHLAK	2	SMC4	803	LQKNVR	3
CAP-D2	522	LNYKNAVR	4	CAP-D2	455	LQEMKDR	5
CAP-D2	522	LNYKNAVR	4	CAP-D2	530	LTQKALCcmR	4
SMC2	968	LQKLLTK	3	SMC2	975	KEKLEK	3
CAP-D2	439	LSCcmSDLAEPLKK	11	CAP-D2	455	LQEMKDR	5
CAP-D2	439	LSCcmSDLAEPLKK	11	CAP-D2	455	LQEMoxKDR	5
CAP-D2	530	LTQKALCcmR	4	CAP-D2	450	KEVQK	1
CAP-D2	530	LTQKALCcmR	4	SMC2	177	KITAHK	1
CAP-D2	530	LTQKALCcmR	4	CAP-D2	521	KLNYK	1
CAP-D2	530	LTQKALCcmR	4	CAP-D2	455	LQEMKDR	5
CAP-D2	530	LTQKALCcmR	4	CAP-D2	455	LQEMoxKDR	5
CAP-D2	530	LTQKALCcmR	4	SMC2	172	MoxYECcmKK	5
CAP-D2	530	LTQKALCcmR	4	SMC2	172	MYECcmKK	5
CAP-D2	530	LTQKALCcmR	4	SMC2	183	TIEK	1

SMC2	439	MDGSYKEDQEAFEAIR	6	SMC2	433	QAEVKK	5
SMC2	439	MoxDGSYKEDQEAFEAIR	6	SMC2	433	QAEVKK	5
SMC2	792	NAQQKLNSAK	5	SMC2	774	ALENKMoxK	5
SMC4	434	NISDATSKK	8	SMC4	443	ELLEKAK	5
SMC4	434	NISDATSKK	8	CAP-H	427	FKPRHK	2
SMC2	511	NPEKNWNPNCcmVK	4	SMC2	562	ILEKGQLK	4
CAP-H	73	QADASLPALSGWTNAQISEHYSACcmIK	5	CAP-G	400	EFIGQQLILIIDCcmMoxDTTEEGGRK	17
SMC4	852	QKELEKVLNSYK	6	SMC4	864	KDYER	1
SMC4	577	QKVEEAK	2	SMC2	566	GQLKHR	4
SMC2	740	QQEDLLALKK	9	SMC2	732	IQQSAYHK	6
SMC2	740	QQEDLLALKK	9	SMC2	438	KMoxDGSYKEDQEAFEAIR	1
SMC2	740	QQEDLLALKK	9	SMC2	750	TIAECcmEETLKK	10
SMC2	740	QQEDLLALKK	9	SMC2	424	YAQQELKTK	7
CAP-G	489	QQIKLAEIK	4	CAP-G	498	VKLFEAK	2
SMC4	1094	RKEELYLK	2	SMC2	209	LKEAR	2
SMC4	1094	RKEELYLK	2	CAP-D2	530	LTQKALCcmR	4
SMC4	1094	RKEELYLKR	8	SMC4	278	GEKLNR	3
CAP-D2	963	SDSSTIK	4	CAP-D2	949	ILREEEKTK	7
CAP-H	476	SILENQNVK	1	CAP-H	469	AALTLSK	6
SMC4	362	SKALKDLEK	2	SMC4	354	LADEMoxKSK	6
SMC4	493	SKMDVAQAELDIYLTR	2	SMC2	411	AATEAKQAQMK	6
SMC4	493	SKMDVAQAELDIYLTR	2	SMC2	411	AATEAKQAQMoxK	6
SMC4	493	SKMoxDVAQAELDIYLTR	2	SMC2	411	AATEAKQAQMK	6
SMC4	493	SKMoxDVAQAELDIYLTR	2	SMC2	411	AATEAKQAQMoxK	6
SMC4	4	STKTSTAAHQK	3	SMC4	278	GEKLNR	3

SMC2	248	STNALKEAQANK	6	SMC2	241	AEEIKDR	5
SMC2	248	STNALKEAQANK	6	SMC2	261	KIFESMAENEK	1
CAP-H	229	TAASFDECcmSTAGIFLT SLR	9	SMC4	311	MoxFKEKNHICcmQY YIYDLQKR	5
SMC4	940	TEKEIGDNEK	3	SMC2	330	LKELIK	2
SMC2	750	TIAECcmEETLKK	10	SMC2	767	KAEEEYK	1
CAP-H	467	TKAALTLSK	2	CAP-G	154	DKFSNVR	2
CAP-H	467	TKAALTLSK	2	SMC2	209	LKEAR	2
CAP-D2	153	TKASGFSWEDEREPLLR	2	CAP-D2	137	SGLVEVDPAGKNK	11
SMC2	691	TSQLEATEK	7	H2A.Z	1	AGGKAGK	4
SMC2	691	TSQLEATEK	7	SMC2	700	ELANLKNMAEK	6
SMC2	691	TSQLEATEKELANLK	9	SMC2	682	DAEDELKIK	7
SMC2	691	TSQLEATEKELANLK	9	SMC2	469	YEEAEQEAHLAKK	12
SMC4	7	TSTAAHQKGR	8	SMC4	278	GEKLNR	3
CAP-G	883	VKDKLCcmQR	4	CAP-G	897	KNLTK	1
SMC2	273	VKELAQQIEETEK	2	SMC4	1005	KEALNIK	1
CAP-H	161	VLGGLGKESAPTK	7	SMC4	130	AQKIR	3
CAP-H	161	VLGGLGKESAPTK	7	SMC2	188	ESKLDEIR	3
CAP-H	161	VLGGLGKESAPTK	7	SMC4	278	GEKLNR	3
CAP-H	161	VLGGLGKESAPTK	7	CAP-H	193	VQTKK	4
SMC4	869	VSEQAGKMESEVK	7	SMC4	864	KDYER	1
SMC4	869	VSEQAGKMoxESEVK	7	SMC4	864	KDYER	1
SMC2	997	YNDLMKK	6	SMC4	286	MVEKEK	4
SMC2	997	YNDLMKK	6	SMC4	284	VKMVEK	2
SMC2	997	YNDLMoxKK	6	SMC4	278	GEKLNR	3
SMC4	1030	YWQKEISK	4	SMC2	997	YNDLMKK	6
SMC4	1030	YWQKEISK	4	SMC2	997	YNDLMoxKK	6
CAP-H	433	SSADSEKENK	1	CAP-H	427	FKPR	2
CAP-G	218	TMoxDVKEAVR	5	CAP-H	427	FKPR	2

SMC2/ SMC4 SUB-COMPLEX

Protein1	Peptide Position 1	Peptide 1	Pep 1 Link pos	Protein2	Peptide Position 2	Peptide 2	Pep 2 Link pos
SMC2	411	AATEAKQAQMK	6	SMC2	422	LKYAQQELK	3
SMC2	411	AATEAKQAQMoxK	6	SMC2	422	LKYAQQELK	2
SMC2	241	AEEIKDR	5	SMC2	248	STNALKEAQANKK	6
SMC2	343	AFTSKEK	5	SMC4	929	NLKK	3
SMC4	448	AKDKEEEKLK	4	SMC4	434	NISDATSKK	8
SMC2	774	ALENKMK	5	SMC2	787	GKEIK	2
SMC2	1028	ALHIAWEKVNK	8	SMC4	278	GEKLNR	3
SMC4	364	ALKDLEK	3	SMC2	885	TKDIK	2
SMC4	364	ALKDLEK	3	SMC2	885	TKDIKAK	5
SMC2	618	AMoxEYVFGTTLVCcmSSMDNAKK	19	SMC2	638	VTFDKR	5
SMC4	130	AQKIR	3	SMC4	278	GEKLNR	3
SMC4	919	AQVAIKTAHR	6	SMC2	343	AFTSKEK	4
SMC4	919	AQVAIKTAHR	6	SMC4	932	KSEDSVLR	1
SMC4	919	AQVAIKTAHR	6	SMC4	929	NLKK	3
SMC2	214	ASYLEYQKMoxTR	8	SMC2	348	EKEIK	2
SMC2	214	ASYLEYQKMoxTR	8	SMC2	209	LKEAR	2
SMC2	214	ASYLEYQKMoxTR	8	SMC2	971	LLTKK	3
SMC2	214	ASYLEYQKMTR	8	SMC2	209	LKEAR	2
SMC4	450	DKEEEKLK	6	SMC4	443	ELLEKAK	5
SMC4	367	DLEKK	4	SMC4	362	SKALK	2
SMC4	521	EALMoxTTSETLKQR	11	SMC4	535	AAIKDIDIK	4

SMC4	521	EALMoxTTSETLKQR	11	SMC4	534	KAAIK	1
SMC4	521	EALMoxTTSETLKQR	11	SMC4	831	LKGQVK	2
SMC4	521	EALMTTSETLKQR	11	SMC4	535	AAIKDIDIK	4
SMC4	521	EALMTTSETLKQR	11	SMC4	534	KAAIK	1
SMC4	521	EALMTTSETLKQR	11	SMC4	831	LKGQVK	2
SMC4	1096	EELYLKR	6	SMC4	278	GEKLNR	3
SMC4	475	EGKEKELMEFCcmK	5	SMC4	469	EIQKEK	4
SMC4	478	EKELMEFCcmK	2	SMC2	779	MKNAEAER	2
SMC4	478	EKELMEFCcmK	2	SMC2	779	MoxKNAEAER	2
SMC4	478	EKELMoxEFCcmK	2	SMC2	779	MKNAEAER	2
SMC4	478	EKELMoxEFCcmK	2	SMC2	779	MoxKNAEAER	2
SMC4	478	EKELMoxEFCcmKTVNDAR	2	SMC2	779	MKNAEAER	2
SMC4	478	EKELMoxEFCcmKTVNDAR	10	SMC2	779	MoxKNAEAER	2
SMC4	398	EKLKHAK	4	SMC2	308	VDAKVR	4
SMC2	458	EKLQDEMK	2	SMC4	534	KAAIK	1
SMC2	700	ELANLKNMAEK	6	SMC4	803	LQKNVR	3
SMC2	700	ELANLKNMoxAEK	6	SMC4	803	LQKNVR	3
SMC2	275	ELAQQIEETEK	9	SMC2	286	KNNEEFGAK	1
SMC4	854	ELEKVLNSYK	4	SMC4	864	KDYER	1
SMC2	332	ELIKIMQEEFK	4	SMC4	940	TEKEIGDNEK	3
SMC4	480	ELMEFCcmKTVNDAR	8	SMC2	779	MoxKNAEAER	2
SMC4	480	ELMoxEFCcmKTVNDAR	8	SMC2	779	MKNAEAER	2
SMC4	480	ELMoxEFCcmKTVNDAR	8	SMC2	779	MoxKNAEAER	2
SMC2	851	EQVSALEAEAVKTR	12	SMC2	865	ESLKNAENELSSEK	4
SMC2	851	EQVSALEAEAVKTR	13	SMC2	308	VDAKVR	4
SMC2	188	ESKLDEIRR	3	SMC4	278	GEKLNR	3
SMC2	865	ESLKNAENELSSEK	4	SMC4	386	FTQLDLQDVKVR	10
SMC2	963	ETGQKLQK	5	SMC2	971	LLTKK	4

SMC2	507	FEYKNPEK	4	SMC2	562	ILEKGQLK	4
SMC4	386	FTQLDLQDVKVR	10	SMC4	398	EKLK	2
SMC4	386	FTQLDLQDVKVR	10	SMC2	865	ESLKNAENELSSEK	4
SMC4	386	FTQLDLQDVKVR	10	SMC2	320	KQNLNSEENR	1
SMC4	738	GEIIEQSGTMTGGGGKVMK	16	SMC4	577	QKVEEAK	2
SMC4	738	GEIIEQSGTMTGGGGKVMoxK	16	SMC4	577	QKVEEAK	2
SMC4	278	GEKLNR	3	SMC4	130	AQKIR	3
SMC4	278	GEKLNR	3	SMC2	188	ESKLDEIRR	2
SMC4	278	GEKLNR	3	SMC4	1095	KEELYLKR	7
SMC4	278	GEKLNR	3	SMC4	1094	RKEELYLKR	8
SMC4	278	GEKLNR	3	SMC2	997	YNDLMoxKK	6
SMC2	566	GQLKHR	4	SMC4	577	QKVEEAK	2
SMC4	372	IDKITVLIEENKNK	3	SMC4	367	DLEKK	4
SMC2	262	IFESMAENEKK	10	SMC2	273	VKELAQQIEETEKK	13
SMC2	197	IITEEISPTLEK	7	SMC2	209	LKEAR	2
SMC2	197	IITEEISPTLEKLK	12	SMC4	278	GEKLNR	3
SMC2	197	IITEEISPTLEKLK	12	SMC4	1095	KEELYLK	1
SMC2	354	IKEGLNGLQEESKK	2	SMC2	348	EKEIK	2
SMC2	354	IKEGLNGLQEESKK	2	SMC2	348	EKEIKK	2
SMC2	562	ILEKGQLK	4	SMC2	507	FEYKNPEK	4
SMC2	336	IMoxQEEFKAFTSK	7	SMC2	348	EKEIK	2
SMC2	336	IMoxQEEFKAFTSK	7	SMC2	348	EKEIKK	5
SMC2	336	IMoxQEEFKAFTSK	7	SMC2	975	KEKLEK	1
SMC2	336	IMoxQEEFKAFTSK	7	SMC2	330	LKELIK	2
SMC2	336	IMQEEFKAFTSK	7	SMC2	348	EKEIK	2
SMC2	336	IMQEEFKAFTSK	7	SMC2	348	EKEIKK	5
SMC2	336	IMQEEFKAFTSK	7	SMC2	975	KEKLEK	1
SMC2	336	IMQEEFKAFTSK	7	SMC2	330	LKELIK	2

SMC2	732	IQQSAYHK	4	SMC2	433	QAEVKK	5
SMC4	534	KAAIK	1	SMC4	831	LKGQVK	2
SMC2	767	KAEEEYK	1	SMC2	774	ALENKMK	5
SMC2	767	KAEEEYK	1	SMC2	774	ALENKMoxK	5
SMC2	767	KAEEEYKALENK	7	SMC2	779	MKNAEAER	2
SMC4	563	KEELNAQDLVR	1	SMC4	553	EKENKLEK	5
SMC4	563	KEELNAQDLVR	1	SMC4	555	ENKLEKLK	6
SMC4	563	KEELNAQDLVR	1	SMC4	558	LEKLK	3
SMC4	1095	KEELYLKR	7	SMC4	278	GEKLNR	3
SMC2	261	KIFESMoxAENEK	1	SMC2	248	STNALKEAQANKK	6
SMC2	353	KIKEGLNGLQEESK	3	SMC2	348	EKEIK	2
SMC2	353	KIKEGLNGLQEESKK	3	SMC2	348	EKEIK	2
SMC2	481	KKQLSSEISSLR	2	SMC4	803	LQKNVR	3
SMC4	675	KNLQK	1	SMC2	637	KVTFDKR	6
SMC2	286	KNNEEFGAK	1	SMC2	275	ELAQQIEETEK	9
SMC2	482	KQLSSEISSLR	1	SMC4	786	AVQCcmEEEKFQLEE DITK	8
SMC2	482	KQLSSEISSLR	1	SMC2	700	ELANLKNMoxAEK	6
SMC2	482	KQLSSEISSLR	1	SMC4	803	LQKNVR	3
SMC2	482	KQLSSEISSLR	1	SMC2	469	YEEAEQEAHLAKK	12
SMC2	320	KQNLNSEENR	1	SMC4	386	FTQLDLQDVKVR	10
SMC4	932	KSEDSVLR	1	SMC4	417	DKEKVEELK	4
SMC4	354	LADEMKSK	6	SMC4	364	ALKDLEK	3
SMC4	354	LADEMKSK	6	SMC4	362	SKALK	2
SMC4	354	LADEMoxKSK	6	SMC4	364	ALKDLEK	3
SMC4	354	LADEMoxKSK	6	SMC4	362	SKALK	2
SMC4	354	LADEMoxKSK	6	SMC4	362	SKALKDLEK	2
SMC4	410	LEKQLQK	3	SMC4	417	DKEKVEELK	4
SMC2	209	LKEAR	2	SMC4	1094	RKEELYLK	2
SMC4	561	LKKEELNAQDLVR	2	SMC4	728	DKRWR	2

SMC4	561	LKKEELNAQDLVR	3	SMC4	553	EKENKLEK	5
SMC4	561	LKKEELNAQDLVR	3	SMC4	555	ENKLEK	3
SMC4	456	LKQVMSSLQEETR	2	SMC2	787	GKEIKNAQQK	5
SMC4	456	LKQVMSSLQEETR	2	SMC2	802	KKADDSSR	1
SMC2	422	LKYAQQELK	2	SMC2	411	AATEAKQAQMK	6
SMC2	422	LKYAQQELK	3	SMC2	411	AATEAKQAQMoxK	6
SMC2	422	LKYAQQELK	3	SMC4	850	NKQK	2
SMC2	467	LKYEEAEQEAHLAK	2	SMC2	706	NMoxAEKYQHLK	6
SMC2	467	LKYEEAEQEAHLAK	2	SMC2	711	YQHLKQQWEMoxK	5
SMC4	544	LPTAEQELKEK	9	SMC4	558	LEKLK	3
SMC2	548	LYNIVVDTEATGK	11	SMC2	561	KILEKGQLK	1
SMC2	548	LYNIVVDTEATGKK	13	SMC2	562	ILEKGQLK	4
SMC2	439	MoxDGSYKEDQEAFEAI RK	6	SMC2	433	QAEVKK	5
SMC2	779	MoxKNAEAER	2	SMC2	767	KAEEEYKALENK	6
SMC2	873	NAENELSSEKGLMAER	10	SMC2	885	TKDIK	2
SMC2	792	NAQQKLNSAK	5	SMC2	787	GKEIK	2
SMC2	792	NAQQKLNSAK	5	SMC2	803	KADDSSR	6
SMC2	792	NAQQKLNSAK	8	SMC2	802	KKADDSSR	2
SMC4	434	NISDATSKK	8	SMC4	448	AKDKEEEKLK	4
SMC2	830	QEQASYKQQSEAAQQAI ASLK	7	SMC2	336	IMoxQEEFKAFTSK	7
SMC4	577	QKVEEAK	2	SMC2	566	GQLKHR	4
SMC4	577	QKVEEAK	2	SMC4	584	SSLAQHR	2
SMC4	17	QPIEEPAGDMoxQVEKQ DGR	14	SMC4	278	GEKLNR	3
SMC4	17	QPIEEPAGDMQVEKQDG R	14	SMC4	130	AQKIR	3
SMC4	17	QPIEEPAGDMQVEKQDG R	14	SMC4	278	GEKLNR	3

SMC2	740	QQEDLLALKK	9	SMC2	424	YAQQELKTK	8
SMC4	1094	RKEELYLKR	8	SMC4	278	GEKLNR	3
SMC2	314	SDLDHR	1	SMC2	308	VDAKVR	4
SMC2	314	SDLDHRK	1	SMC2	308	VDAKVR	4
SMC2	4	SIVLEGFKSYAQR	8	SMC4	130	AQKIR	3
SMC4	362	SKALKDLEK	2	SMC4	354	LADEMKSK	6
SMC4	493	SKMDVAQAELDIYLTR	2	SMC2	411	AATEAKQAQMK	6
SMC4	493	SKMDVAQAELDIYLTR	2	SMC2	411	AATEAKQAQMoxK	3
SMC4	493	SKMoxDVAQAELDIYLT R	2	SMC2	411	AATEAKQAQMoxK	3
SMC4	351	SSKLADEMK	3	SMC4	343	ETKDINEK	3
SMC4	351	SSKLADEMK	3	SMC4	360	SKSK	3
SMC4	584	SSLAQHR	2	SMC4	754	VMKGR	3
SMC4	584	SSLAQHR	2	SMC4	754	VMoxKGR	3
SMC2	248	STNALKEAQANK	6	SMC2	241	AEEIKDR	5
SMC2	248	STNALKEAQANKK	12	SMC2	241	AEEIKDR	5
SMC4	1270	SVATNPK	1	SMC4	1264	THNTTK	5
SMC2	885	TKDIKAK	5	SMC4	364	ALKDLEK	3
SMC2	885	TKDIKAK	5	SMC2	895	IEKYR	3
SMC2	1158	TKFVDGISTVSR	2	SMC4	130	AQKIR	3
SMC2	1158	TKFVDGISTVSR	2	SMC4	278	GEKLNR	3
SMC2	1158	TKFVDGISTVSR	1	SMC2	209	LKEAR	2
SMC4	897	TQQDKVDKINQEIDECc mTSAITK	8	SMC4	448	AKDKEEEK	4
SMC4	897	TQQDKVDKINQEIDECc mTSAITK	8	SMC4	434	NISDATSKK	8
SMC2	691	TSQLEATEKELANLK	9	SMC2	467	LKYEEAEQEAHLAKK	14
SMC2	691	TSQLEATEKELANLK	9	SMC2	469	YEEAEQEAHLAKK	12
SMC2	308	VDAKVR	4	SMC4	400	LKHAK	2
SMC4	695	VEDKSFSPAFYFALR	7	SMC4	675	KNLQK	1

SMC2	273	VKELAQQIEETEK	11	SMC2	286	KNNEEFGAK	1
SMC2	273	VKELAQQIEETEKK	2	SMC4	1005	KEALNIK	1
SMC4	284	VKMVEK	2	SMC4	278	GEKLNR	3
SMC4	869	VSEQAGKMESEVK	10	SMC4	473	EKEGKEK	2
SMC4	869	VSEQAGKMESEVK	7	SMC4	864	KDYER	3
SMC4	869	VSEQAGKMESEVKR	7	SMC4	473	EKEGK	2
SMC4	869	VSEQAGKMESEVKR	13	SMC4	864	KDYER	1
SMC4	869	VSEQAGKMoxESEVK	10	SMC4	864	KDYER	1
SMC4	869	VSEQAGKMoxESEVKR	13	SMC4	475	EGKEK	3
SMC4	869	VSEQAGKMoxESEVKR	13	SMC4	864	KDYER	1
SMC2	424	YAQQELKTK	8	SMC2	740	QQEDLLALKK	9
SMC2	997	YNDLMKK	6	SMC4	278	GEKLNR	3
SMC2	997	YNDLMoxKK	6	SMC4	278	GEKLNR	3
SMC2	997	YNDLMoxKK	6	SMC4	1030	YWQKEISK	4
SMC4	509	YNTAVSQLDEAKEALMoxTTSETLK	12	SMC4	831	LKGQVK	2
SMC2	711	YQHLKQQWEMoxK	5	SMC4	803	LQKNVR	3
SMC2	919	YQQETADASSTLDKLLK	14	SMC2	936	EYKWIASEK	3
SMC4	817	YTASIQSFSEQEIR	1	SMC4	812	NTLEK	2
SMC2	572	YTIIPLSK	1	SMC4	577	QKVEEAK	2
SMC4	1030	YWQKEISK	4	SMC2	997	YNDLMKK	6
SMC4	1030	YWQKEISK	7	SMC2	997	YNDLMoxKK	6

IN SITU LINKAGES

Protein1	Peptide Position 1	Peptide 1	Pep 1 Link pos	Protein2	Peptide Position 2	Peptide 2	Pep 2 Link pos

CAP-H	2	AVTPR	3	CAP-H	0	MRAVTPRRGGR	1
CAP-G	248	VKLLQQGLNDR	2	CAP-G	210	TLPKIVGR	4
SMC4	386	FTQLDLQDVKVR	10	SMC2	320	KQNLNSEENR	1
SMC4	532	QRKAAIKDIDIK	7	SMC2	566	GQLKHR	4
SMC4	78	LMoxITHIVNQNFK	4	SMC4	90	SYAGEQTLGPFHK	2
SMC4	897	TQQDKVDKINQEIDECcmTSAITK	5	SMC4	434	NISDATSKK	8
SMC4	695	VEDKSFSPAFYFALR	4	SMC4	676	NLQKIPTPENAPR	7
SMC2	197	IITEEISPTLEKLK	12	CAP-H	161	VLGGLGKESAPTK	2
SMC2	411	AATEAKQAQMK	6	SMC2	422	LKYAQQELK	4
SMC2	507	FEYKNPEK	4	SMC2	562	ILEKGQLK	4
SMC2	792	NAQQKLNSAK	5	SMC2	787	GKEIK	2
SMC2	424	YAQQELKTK	7	SMC2	740	QQEDLLALKK	2
SMC2	197	IITEEISPTLEK	9	SMC2	209	LKEAR	10
SMC2	865	ESLKNAENELSSEK	4	SMC4	386	FTQLDLQDVKVR	1
Histone H4	80	TVTAMDVVYALK	3	CAP-D2	1380	RTALR	2
Histone H4	24	DNIQGITKPAIR	8	CAP-D2	1380	RTALR	2
Histone H4	80	TVTAMoxDVVYALKR	12	Histone H2A-III	36	KGNYAER	1
Histone H4	56	GVLKVFLENVIR	4	Histone H2A-III	4	GKQGGK	2
Histone H4	24	DNIQGITKPAIR	8	Histone H4	24	DNIQGITKPAIR	7
Histone H4	24	DNIQGITKPAIR	8	Histone H4	6	GGKGLGK	3
Histone H4	24	DNIQGITKPAIR	8	Histone H4	37	LARRGGVKR	8
Histone H4	24	DNIQGITKPAIR	8	Histone H4	9	GLGKGGAK	4

Histone H4	24	DNIQGITKPAIR	8	Histone H4	13	GGAKR	4
Histone H4	56	GVLKVFLENVIR	4	Histone H4	6	GGKGLGK	3
Histone H4	56	GVLKVFLENVIR	4	Histone H4	4	GKGGK	2
Histone H4	56	GVLKVFLENVIR	4	Histone H4	9	GLGKGGAK	4
Histone H4	79	KTVTAMDVVYALK	1	Histone H4	6	GGKGLGK	3
Histone H4	80	TVTAMDVVYALKR	12	Histone H4	41	GGVKR	4
Histone H2A-III	100	VTIAQGGVLPNIQAVLLPKK	19	Histone H2A-III	120	TDSHK	1
Histone H2A-III	4	GKQGGK	2	Histone H2A.Z	5	AGKDSGK	3
Histone H2A-III	89	NDEELNKLLGK	7	Histone H4	9	GLGKGGAK	4
Histone H2A-III	89	NDEELNKLLGK	7	Histone H4	6	GGKGLGK	3
Histone H2A-III	89	NDEELNKLLGK	7	Histone H4	4	GKGGK	2
Histone H2A-III	100	VTIAQGGVLPNIQAVLLPKK	19	Histone H4	13	GGAKR	4
Histone H2A-III	100	VTIAQGGVLPNIQAVLLPKK	19	Histone H4	41	GGVKR	4
Histone H2A-III	100	VTIAQGGVLPNIQAVLLPKK	19	SMC2	58	ASSLQDLVYK	9
Histone H2A-III	100	VTIAQGGVLPNIQAVLLPKK	19	SMC4	605	SGSISGIHGR	3

COHESIN COMPLEX

Protein1	Peptide Position 1	Peptide 1	Pep 1 Link pos	Protein2	Peptide Position 2	Peptide 2	Pep 2 Link pos
SMC3	308	AKDLQDELAGNSEQR	2	SMC1	294	RPQYIKAK	6
SMC3	236	AKLDELSAK	2	SMC1	968	SSSLYAR	5
SMC3	236	AKLDELSAKR	9	SMC3	246	ETSGEKSR	6
RAD21	70	AKYLLADCcmNEAFIK	2	SMC3	186	EKINELLK	2
SMC1	218	AQVQLQLFKLYHNEAEIEK	9	SMC1	918	LEQKR	4
RAD21	390	CcmLTPLVPEDLR	3	RAD21	383	LLKLFTR	3
SMC1	554	DCcmIQYIKEQR	7	SMC3	661	GALTGGYYDTR	7
SMC1	62	DLIHGAPVGKPASNR	10	SMC1	96	LIVGSSSEYKINNR	10
SMC1	62	DLIHGAPVGKPASNR	10	SMC1	13	SYKGR	3
SMC1	62	DLIHGAPVGKPASNR	10	SMC1	57	VKALR	2
SMC1	363	DLTLEENQVKK	10	SMC1	377	LKEEASKR	7
SMC3	15	DQTIVDPFSSKHNVIVGR	11	SMC3	0	MoxYIK	1
SMC3	15	DQTIVDPFSSKHNVIVGR	11	SMC3	1099	VSFTGKQGEMoxR	6
SMC3	397	ELKSLDQAINDK	3	SMC3	391	DKWIKK	5
SMC3	895	ELQKSMoxER	4	SMC1	255	MoxDRVEDELKDR	9
SMC1	338	EMGAVEKAR	7	SMC1	807	LEFENQKTR	7
SMC1	338	EMoxGAVEKAR	7	SMC1	807	LEFENQKTR	7
SMC1	173	EMoxVKAEEDTQFNYHR	4	SMC1	160	SGELAQEYDKR	10
SMC1	910	EVTAIETKLEQK	8	SMC3	928	QGMoxLLKK	6
SA-2	976	FALTFGLDQLKTR	11	SA-2	930	QIDKIQCcmAK	4

SMC3	661	GALTGGYYDTR	7	SMC1	554	DCcmIQYIKEQR	7
SMC1	858	HMoxKIIDETMAQLQDLK	3	SMC1	853	KEEQR	1
SMC1	858	HMoxKIIDETMoxAQLQDLK	3	SMC1	853	KEEQR	1
SMC1	858	HMoxKIIDETMoxAQLQDLK	3	SMC1	851	LKKEEQR	3
SMC1	434	IEKLEEYIATSK	3	SMC1	426	EIEENQKR	7
SMC3	818	IKLEGIITR	2	SMC3	355	EKEER	2
SMC3	818	IKLEGIITR	2	SMC3	336	IEEKQK	4
RAD21	84	IKMAFRPGVVDLPEENR	2	SMC3	186	EKINELLK	2
RAD21	84	IKMoxAFRPGVVDLPEENR	2	SMC3	186	EKINELLK	2
SA-2	769	ITESSSTKEDLLR	8	SA-2	784	KQMoxR	1
SMC3	683	KAEEELGELEAK	1	SMC3	492	KQQLLR	1
SMC3	683	KAEEELGELEAK	1	SMC3	675	LELQKDVR	5
SMC3	683	KAEEELGELEAK	1	SMC3	673	SRLELQKDVR	7
SMC3	683	KAEEELGELEAKLNENLR	12	SMC3	474	EENAEQQALAAKR	12
SMC3	984	KALDQFVNFSEQK	1	SMC1	211	LKDEVVR	2
SMC1	843	KDEAEIEK	1	SMC1	302	ENTAHKIK	6
SMC1	843	KDEAEIEKLK	8	SMC1	853	KEEQR	1
SMC3	171	KEESISLMoxK	1	SMC1	172	KEMoxVK	1
SMC3	171	KEESISLMoxK	1	SMC3	155	LKLLR	2
SMC3	1058	KGDVEGSQSQDEGEGSTESER	1	SMC3	1024	KYEAIQLTFKQVSK	10
RAD21	404	KGGEADNLDEFLK	1	RAD21	383	LLKLFTR	3
RAD21	404	KGGEADNLDEFLK	1	RAD21	383	LLKLFTR	3
SMC1	412	KKVETEAK	2	SMC1	747	INDIKR	5
RAD21	321	KLIVDSVK	1	RAD21	329	ELDSKTIR	5

SA-2	575	KTQLDDR	1	SA-2	568	VLTAKEK	5
SA-2	575	KTQLDDRTK	1	SA-2	568	VLTAKEK	5
SMC1	413	KVETEAK	1	SMC1	747	INDIKR	5
SMC3	453	KYYEVKNKKDELQSER	9	SMC1	268	ELGKMMoxR	4
SMC3	436	LDQDLNEVKAR	9	SMC3	740	MoxLKEK	3
SMC1	807	LEFENQKTR	7	SMC1	338	EMoxGAVEKAR	7
SMC1	807	LEFENQKTR	7	SMC3	336	IEEKQK	4
SMC1	807	LEFENQKTR	7	SMC3	818	IKLEGIITR	2
SA-2	626	LEKHLDALLR	3	SA-2	563	GTGKR	4
SMC3	675	LELQKDVR	5	SMC3	492	KQQLLR	1
SMC1	5	LIEIENFKSYK	8	SA-2	276	IIGKR	4
SMC1	211	LKDEVVR	2	SMC1	934	MoxQDIKLPLSK	5
SMC1	211	LKDEVVR	2	SMC3	978	YSHVNKK	6
SMC1	377	LKEEASKR	2	SMC1	763	DLKEK	3
SMC1	377	LKEEASKR	7	SMC1	373	KYHR	1
SMC3	155	LKLLR	2	SMC1	57	VKALR	2
RAD21	383	LLKLFTR	3	RAD21	329	ELDSKTIR	5
SMC3	1046	LVPGGKATLVMK	6	SMC3	1099	VSFTGKQGEMoxR	6
SMC1	227	LYHNEAEIEKLNK	10	SMC1	918	LEQKR	4
SMC1	255	MoxDRVEDELKDR	9	SMC1	275	EQQQIEKEIK	7
SMC1	934	MoxQDIKLPLSK	5	SMC1	211	LKDEVVR	2
SMC1	934	MQDIKLPLSK	5	SMC1	211	LKDEVVR	2
SMC3	459	NKKDELQSER	2	SMC3	453	KYYEVK	1
SMC3	295	QEQIKQR	5	SMC1	300	AKENTAHK	2
SMC3	295	QEQIKQR	5	SMC3	304	LELKAK	4
SMC3	295	QEQIKQR	5	SMC3	302	TKLELK	2
SMC3	140	QGKINQMATAPDSQR	3	SMC3	155	LKLLR	2
SMC3	140	QGKINQMoxATAPDSQR	3	SMC3	155	LKLLR	2
SA-2	461	RDPEDDGILKR	10	SA-2	456	KLFSR	1
SA-2	461	RDPEDDGILKR	10	SA-2	626	LEKHLDALLR	3

SMC3	838	RLDQVEQELNELRETEGGTVLTATTSELEAINKR	33	SMC3	872	VKDTLAR	2
SMC1	806	RLEFENQKTR	8	SMC3	818	IKLEGIITR	2
SMC1	294	RPQYIKAK	6	SMC3	872	VKDTLAR	2
SMC1	160	SGELAQEYDKR	10	SMC1	171	KKEMoxVK	2
SMC1	160	SGELAQEYDKR	10	SMC1	171	KKEMVK	2
SMC1	160	SGELAQEYDKRK	10	SMC1	247	EIDKDKKRMDR	4
SMC1	160	SGELAQEYDKRK	10	SMC1	172	KEMoxVK	1
SMC1	160	SGELAQEYDKRK	10	SMC1	172	KEMVK	1
SMC1	734	SKLESELANFGPR	2	SMC3	427	EKNLEQYSK	2
SMC1	734	SKLESELANFGPR	2	SMC1	412	KKVETEAK	2
SMC1	968	SSSLYAR	5	SMC3	236	AKLDELSAK	2
SMC3	12	SYRDQTIVDPFSSKHNVIVGR	14	SMC3	0	MoxYIK	1
SMC3	302	TKLELKAK	6	SMC3	295	QEQIKQR	5
SMC3	827	VETYLNENLR	4	SMC1	807	LEFENQKTR	7
SMC3	872	VKDTLAR	2	SMC1	294	RPQYIKAK	6
SMC3	1099	VSFTGKQGEMoxR	6	SMC1	62	DLIHGAPVGKPASNR	10
SMC3	1099	VSFTGKQGEMoxR	6	SMC1	57	VKALR	2
SMC3	1099	VSFTGKQGEMR	6	SMC3	1046	LVPGGKATLVMoxK	6
SMC3	166	VYDERKEESISLMoxK	6	SMC3	155	LKLLR	2
