# Supplementary material for: Three-dimensional topology of the SMC2/SMC4 subcomplex from chicken condensin I revealed by cross-linking and molecular modelling
Source: Open Biol. 2015 Feb 25;5(2):150005. doi: 10.1098/rsob.150005 (PMC4345284; doi:10.1098/rsob.150005)
Supplement: Barysz_et_al_Supplementary Data File 1 [file rsob150005supp4.zip › Barysz_et_al.VIEWING_INSTRUCTIONS_README.pdf]

VIEWING INSTRUCTIONS for contents of folder "chickenSMC2SMC4core.Barysz_et_al_2015"

Supplementary Data File 1 accompanying Barysz et al. (2015), in review.
Low-resolution model of a full-length rod-like chicken SMC2-SMC4 core complex.

Upon uncompressing, the resulting folder "chickenSMC2SMC4core.Barysz_et_al_2015" includes the atomic 3D coordinates (PDB format) (file name: Barysz_et_al_2015.chickenSMC2SMC4_draft_struc.pdb), the same model pre-rendered for easy visualization for UCSF Chimera (file name: Barysz_et_al_2015.chickenSMC2SMC4_draft_struc.py), and again pre-rendered for PyMOL (file name: Barysz_et_al_2015.chickenSMC2SMC4_draft_struc.pse), and instructions for downloading the programs, accessing and opening the pre-rendered files to view the structure interactively. **Pre-rendered colors and graphics are as in Figure 8A in the Barysz et al.**

Citation: If you use the model to inform your research please cite the accompanying publication.
Barysz H, Kim JH, Chen ZA, Hudson DF, Rappsilber J*, Gerloff DL*, Earnshaw WC* (2015).
3D topology of a core SMC2/SMC4 sub-complex of chicken condensin I revealed by cross-linking and molecular modelling. *xxx manuscript under review xxx*

---

(0) Double-check that the folder has uncompressed

Most likely the folder name on your computer is “chickenSMC2SMC4core.Barysz_et_al_2015”.
This indicates that your computer has uncompressed the file automatically, when you downloaded it – if this is correct please proceed to (1) or (2).

If instead you see “chickenSMC2SMC4core.Barysz_et_al_2015.tgz”, please either apply a decompression tool (on PC or Mac, e.g. RAR Expander or Stuffit), or uncompress per commandline (UNIX/LINUX systems users try `tar –xvzf chickenSMC2SMC4core.Barysz_et_al_2015.tgz`).

Your folder should contain the three items specified in the legend (above), and this instruction file.

(1) Experts

If you have experience with viewing protein 3D-structures you may have a preferred viewing program. If this is not UCSF Chimera or PyMOL, please use the .pdb file. While we have not tested other programs, it adheres to the standards and should open in any standard viewing program for PDB. For convenience we have included pre-rendered sessions for UCSF Chimera (recommended) and PyMOL. Downloading and intro tutorial sites are provided below.

(2) Non-Experts

If you do not have any experience you have two easy options to view this model on your computer (and manipulate it if you like) in only a few steps. You will need to install one of the two programs “UCSF Chimera” or “PyMOL”, on your computer, then you can open a pre-rendered version of the model and take it from there. (There are other easy programs, some of them online – however, due to the large size of this model, we strongly recommend that you consider Chimera or PyMOL, it is worth the small extra effort!)

See below for where to find download and instruction sites by the developers of these programs. Note also that – at the time of writing - PyMOL is only free for academic educators (look for the Educational version or email them), and available for an (affordable) licensing fee. UCSF Chimera is free for all. Please double-check that this information is accurate, when you download the programs!

UCSF Chimera – quick instructions (we recommend the TUTORIAL SITE LINKS below beforehand!)

- launch the program on your computer
- use the pull-down menus to view the structure:
  File > Restore Session…
  [then find the Chimera session file Barysz_et_al_2015.chickenSMC2SMC4_draft_struc.py]
- to be sure you can work properly also open a commandline (appears at the bottom) (optional):
  Tools > General Controls > Command line
- use your mouse to see labels for individual residues (hover over the graphic)
- and/or display the sequences (in separate windows; only modelled residues are shown):
  Tools > Sequence > Sequence

PyMOL (Schrödinger LLC) – quick instructions (recommended if you already have the program)

- launch the program on your computer
- use the pull-down menus to view the structure:
  File > Open…
  [then find the PyMOL session file Barysz_et_al_2015.chickenSMC2SMC4_draft_struc.pse]
- if you don't see the sequence displayed at the top (only modelled residues are shown), make it appear:
  Display > Sequence
- to identify individual residues click on them to select them (in the graphic or the sequence)

(3) Download and Tutorial sites

UCSF Chimera:

- Download: http://www.cgl.ucsf.edu/chimera/download.html
- Beginner's Tutorial by Chimera's developers: http://www.cgl.ucsf.edu/Outreach/Tutorials/GettingStarted.html
- Video Tutorial (unrelated to this project) at the Protein Data Bank (PDB) that includes/uses UCSF Chimera – **be sure to scroll down in the page to Part 2, this helps for quickly learning Chimera use:** http://www.rcsb.org/pdb/101/static101.do?p=education_discussion/educational_resources/videochallenge/video_challenge_tutorial-1.html
- Citation information: http://www.cgl.ucsf.edu/chimera/docs/credits.html

PyMOL (Schrödinger, LLC):

- Download & Licensing: http://www.pymol.org
- Education-only version: http://pymol.org/educational/
- Beginner's Tutorial*: http://www.pymolwiki.org/index.php/Practical_Pymol_for_Beginners
- Citation information: http://www.pymol.org/citing

*: you will likely find more in a web search, many school/university teachers have produced some.

This instruction documentation was written and deemed accurate at time of writing,
by Dietlind L Gerloff on January 8, 2015.
